# Artificial Intelligence Based Skin Analysis Models for Predicting Visual Grades and Device Measured Physiological Values From Facial Images

**DOI:** 10.1111/srt.70375

**Published:** 2026-08-31

**Authors:** Eunyoung Lee, Jeongho Lee, Nahee Kim, Junchae Na, Byungcheol Park, Sang‐Il Choi

**Affiliations:** ^1^ Institut d'Expertise Clinique (IEC) Korea Suwon South Korea; ^2^ Department of Computer Engineering Dankook University Yongin Gyeonggi‐do South Korea; ^3^ Kailoslab Co., Ltd Seoul South Korea; ^4^ Department of Dermatology Dankook University Cheonan Chungcheongnam‐do South Korea

**Keywords:** artificial intelligence, multi‐devices, physiological measurements, visual grading

## Abstract

**Objective:**

This study aimed to develop and validate an artificial intelligence (AI)–based skin assessment framework capable of predicting both dermatologist‐assigned visual grades and device‐derived physiological measurements from facial images. In addition, we evaluated the impact of image‐acquisition modality (DSLR, tablet, or smartphone) on model performance.

**Methods:**

A total of 1,099 Korean participants aged 14–69 years were enrolled. High‐resolution facial images were obtained from seven standardized angles using a DSLR camera, and a subset of additional images was collected using a tablet and participant‐owned smartphones. Five board‐certified dermatologists graded eight facial signs, and physiological parameters were measured using non‐invasive skin assessment devices. CoAtNet‐4 served as the backbone architecture for both classification (visual grading) and regression (physiological prediction). Model performance was assessed using mean absolute error and correlation analyses.

**Results:**

When evaluated using DSLR images, the model achieved a mean exact‐grade accuracy of 51.4%, with an average of 93.3% of predictions falling within ± 1 grade across facial signs. High correlations were observed for wrinkles, pigmentation, pores, and sagging, whereas lip dryness demonstrated comparatively lower correlations. For physiological metrics, strong correlations were observed for pigmentation spot count, cheek pore visibility, and wrinkle severity, whereas hydration and elasticity showed moderate correlations. Performance on mobile‐device images remained high and showed strong agreement with DSLR‐based predictions, although a noticeable decline in pigmentation‐related accuracy was observed.

**Conclusion:**

The proposed AI framework reliably approximates dermatologist visual grading and multiple device‐based physiological measurements, offering a comprehensive, image‐driven approach to skin aging assessment. However, model performance varies across facial attributes and remains sensitive to image quality, emphasizing the need for domain adaptation and image enhancement strategies to ensure robust application in consumer environments.

## 1 Introduction

The skin is one of the most prominent indicators of chronological and biological aging. Skin aging is a gradual and multifactorial process influenced by intrinsic factors—such as genetic predisposition, ethnicity, cellular senescence, and oxidative stress—as well as extrinsic factors including ultraviolet (UV) exposure, environmental pollution, and lifestyle habits [1]. As aging progresses, the structural integrity of the skin barrier deteriorates, accompanied by a reduction in the production of collagen and elastin in the dermis, impaired barrier function, decreased lipid synthesis, and diminished moisture retention. These physiological changes manifest as visible signs of aging, such as reduced elasticity, sagging, increased wrinkling, dryness, uneven skin tone, hyperpigmentation, age spots, and enlarged pores [2, 3].

In dermatological and medical research, skin conditions can be assessed through both visual inspection and instrumental analysis. Visual assessment enables a comprehensive evaluation of external features, such as wrinkles and pigmentation, but it is inherently subjective and susceptible to inter‐evaluator variability [4, 5]. In contrast, instrumental analysis provides objective and reproducible measurements of parameters that are difficult to assess visually, such as moisture content and elasticity. However, current instruments are limited in their ability to deliver a holistic assessment of skin condition [6, 7]. As such, the two approaches are complementary and are often used in combination depending on the purpose of the evaluation.

Recent advances in artificial intelligence (AI) and deep learning have spurred active research into skin condition prediction using facial images in both medical and cosmetic contexts [8, 9]. Most of the latest AI‐based skin imaging diagnostic techniques aim to serve as alternatives to visual assessment [10, 11, 12]. Nevertheless, to provide a comprehensive diagnosis of diverse skin conditions, it is essential to analyze both visually detectable features and those that are not readily discernible.

With the evolution of deep learning methods, convolutional neural networks (CNNs) have been widely applied to image‐based skin analysis. The improved reliability of image‐based evaluations driven by these technologies has further accelerated research in this field [13, 14]. While CNNs are particularly effective at capturing localized skin features, they are limited in their ability to assess overall skin condition. In contrast, Transformer architectures, which leverage the self‐attention mechanism [15], are capable of modeling global patterns within an image, thereby addressing the limitations of CNNs.

In this study, we propose a novel skin condition assessment framework based on CoAtNet‐4 [16], a hybrid deep learning model that combines the strengths of CNNs and Transformers. This model enables the simultaneous learning of local features and global relationships in skin images. We aim to evaluate whether the proposed model can predict not only visually assessed features but also values obtained through instrumental measurements, thereby enabling more precise and comprehensive assessment of skin condition.

## 2 Materials and Methods

### 2.1 Study Population

A total of 1,099 Korean male and female participants, aged between 14 and 69 years, were recruited with an even distribution across adult age groups, excluding individuals in their teens (see Table 1). The study protocol was approved by the Institutional Review Board (IRB) of IEC KOREA (Approval No.: IECK(1)‐IRB‐202307‐102‐06) and was conducted by the guidelines of the International Council for Harmonisation of Technical Requirements for Pharmaceuticals for Human Use (ICH), Good Clinical Practice (GCP), the Declaration of Helsinki, and its subsequent revisions. All participants provided written informed consent after receiving a thorough explanation of the study procedures, including consent for the publication of the study findings.

**TABLE 1:** Distribution of participants. N indicates the number of participants in each age group.

	Age Mean±SD (N)	
Age class, years	Female	Male
14–19	15.24 ± 1.99 (55)	15.51 ± 1.98 (55)
20–29	24.69 ± 2.53 (99)	25.18 ± 2.55 (99)
30–39	35.31 ± 3.09 (99)	34.06 ± 3.20 (99)
40–49	44.30 ± 3.11 (99)	44.19 ± 2.87 (99)
50–59	54.26 ± 2.74 (99)	54.70 ± 3.16 (99)
60–69	63.84 ± 2.37 (99)	63.83 ± 2.71 (98)
Total	41.56 ± 15.97 (550)	41.46 ± 15.99 (549)

### 2.2 Facial Skin Image Dataset

#### 2.2.1 Image Acquisition

**FIGURE 1:**
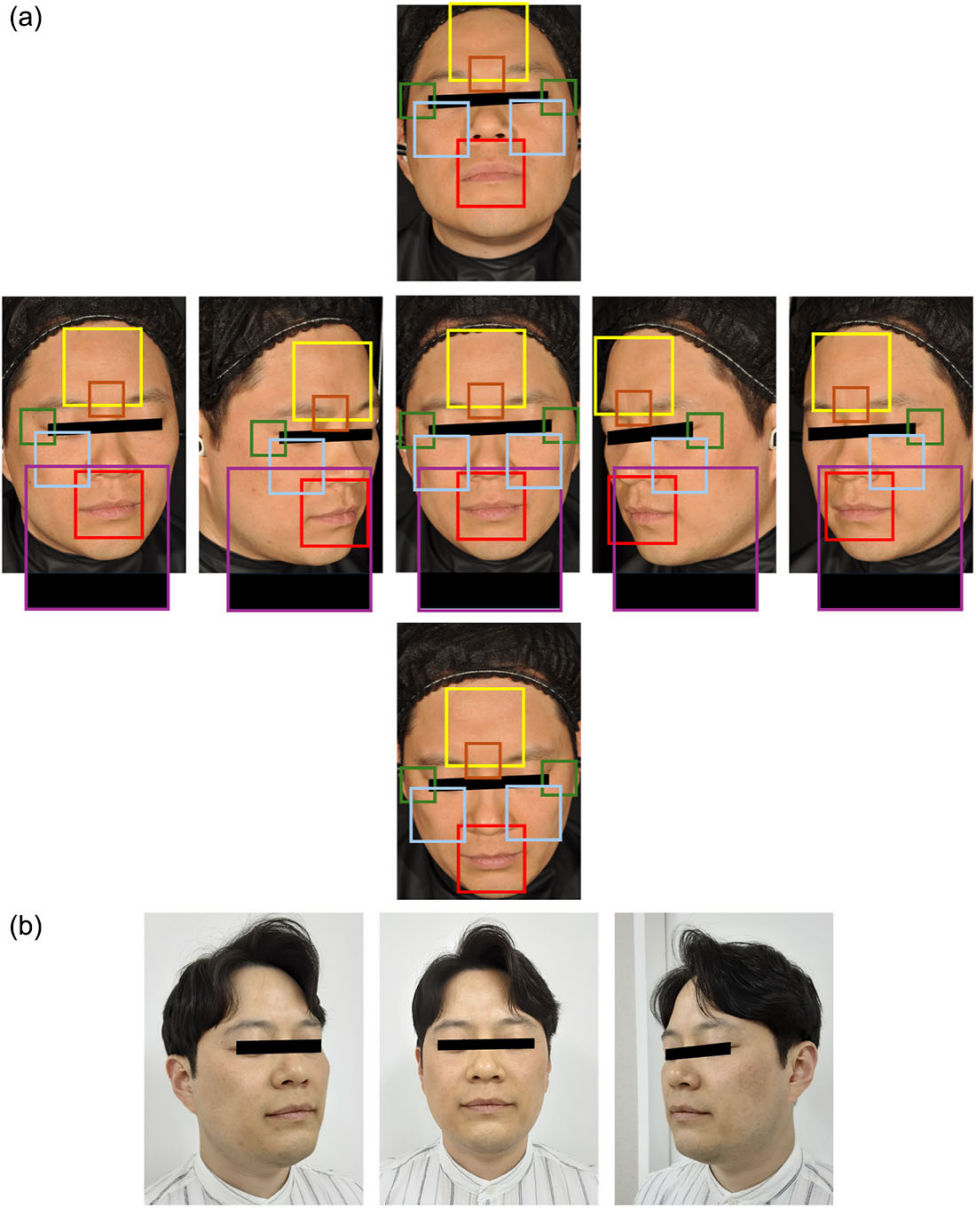
(a) Example facial images captured from seven angles with facial landmarks overlaid. (b) Example facial images captured from three angles using a mobile device.

Figure 1(a) presents DSLR images captured from seven viewing angles with facial landmarks overlaid, whereas Figure 1(b) presents mobile‐device images captured from three viewing angles. Before image acquisition, all participants removed any makeup, gently wiped their faces with tissue after cleansing, and rested for 30 min in a controlled environment maintained at 22 ± 2°C and 50 ± 5% relative humidity. To capture standardized the facial images, we constructed a custom photo booth equipped with a Nikon D2X (Nikon Corporation, Tokyo, Japan) digital single‐lens reflex (DSLR) camera and a flash strobe, as depicted in Figure 1. For each participant, images were captured from seven angles relative to the frontal plane: front, 15° left, 15° right, 30° left, 30° right, upward, and downward. A total of 7,693 facial images (1,099 participants × 7 images per participant) were collected using the DSLR and used in this study. In addition, for comparative analysis under conditions simulating typical consumer self‐imaging behavior, mobile device images were also acquired from all participants using participants’ personal smartphones and a tablet device (Samsung Galaxy Tab A8), as depicted in Figure 1(b). For each mobile device, images were collected from three angles: frontal, left oblique, and right oblique views. For smartphone image acquisition, participants captured frontal images in their usual selfie‐taking manner with the arm fully extended. For oblique images, the camera position was kept constant while participants rotated only their head to the left or right, guided by markers placed at approximately 45° on either side. For tablet‐based image acquisition, the built‐in grid display was used to improve positional consistency across participants. Facial features were aligned to the grid during frontal capture, and for oblique images, the vertical grid line was aligned with the philtrum to standardize facial positioning.

#### 2.2.2 Data Preprocessing

All facial images were de‐identified to ensure participant privacy. The facial regions analyzed varied depending on the type of visual or physiological skin assessment, including pigmentation, pores, wrinkles, dryness, and sagging. To identify specific facial areas, we utilized MediaPipe [17] to extract facial landmarks (shown as red dots in Figure 1(b)). Based on these landmarks, key facial regions—including the Crow's feet area, glabella, forehead, cheeks, lips, and chin—were automatically detected. Since some landmarks were occasionally occluded depending on the facial angle, manual correction was applied based on the initial landmark detection results to ensure accurate region cropping. For each skin assessment category, a dataset was constructed using the corresponding cropped facial regions. To avoid distortion of elongated facial regions, bounding box–based square cropping was applied before resizing. The overall procedure is illustrated in Figure S1.

#### 2.2.3 Ground Truth Generation for Skin Quality Assessment

Table 2 presents the facial regions evaluated for each skin condition category, indicating whether a visual or physiological assessment was performed, along with the corresponding grading scales or measurement ranges.

**TABLE 2:** Visual assessment grading scales and definitions for facial signs.

Facial sign	Definition	Scales	Visual
Forehead wrinkles	Depth, number, and length of the transverse wrinkles on the forehead	0–6	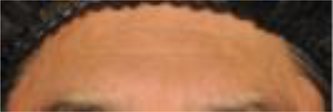
Crow's feet wrinkles	Depth, number, and length of wrinkles at the outer corners of the eyes	0–6	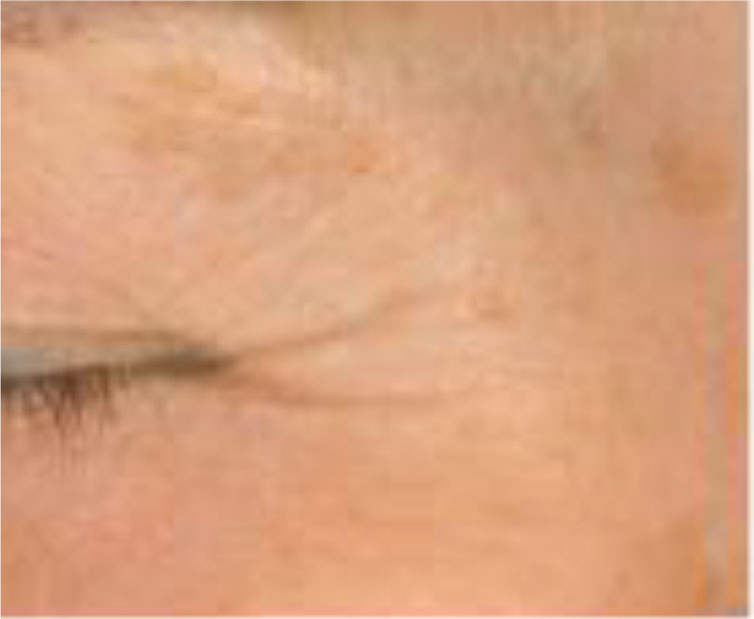
Glabellar wrinkles	Depth, number, and length of vertical wrinkles between the eyebrows	0–6	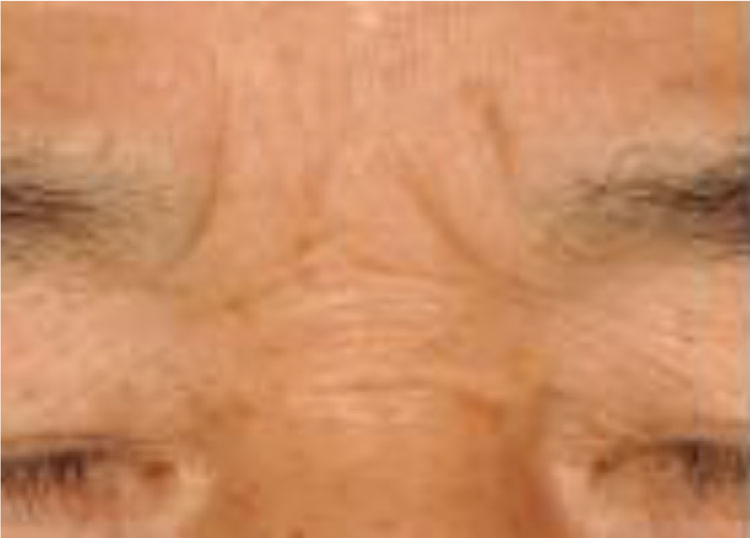
Forehead pigmentation	Hyperpigmentation of the forehead	025	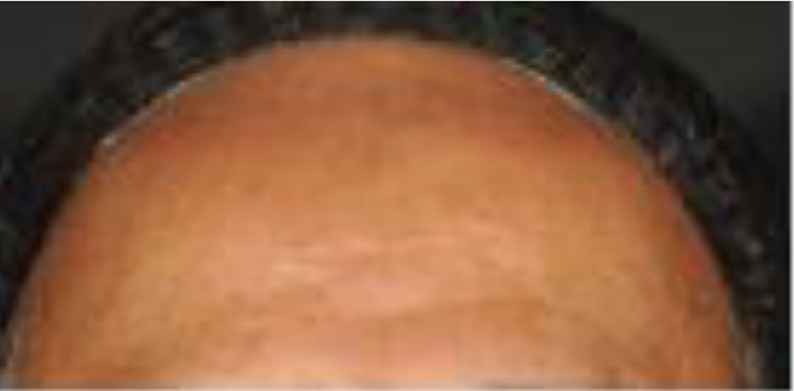
Cheek pigmentation	Severity of spots on the cheekbone area based on number, contrast, and size	0–5	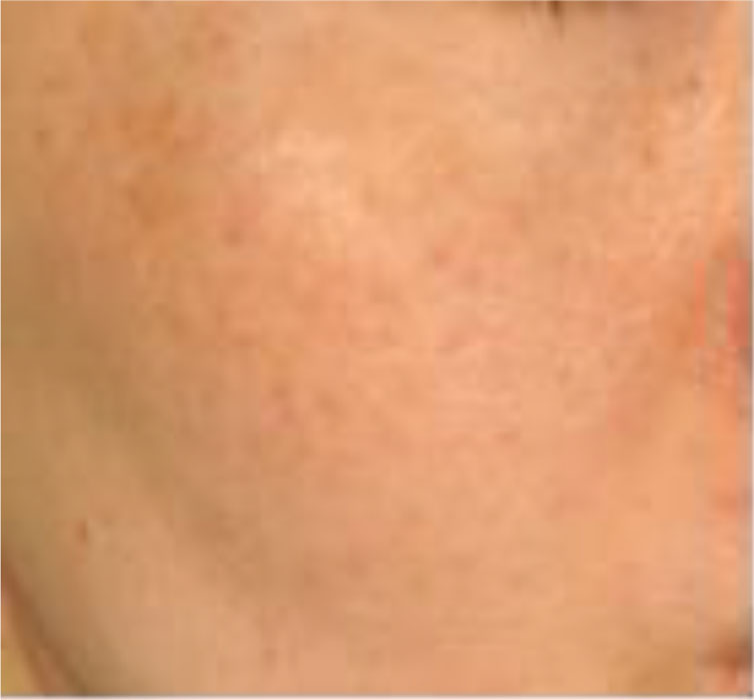
Cheek pores	Size and number of visible pores on the cheek	0–5	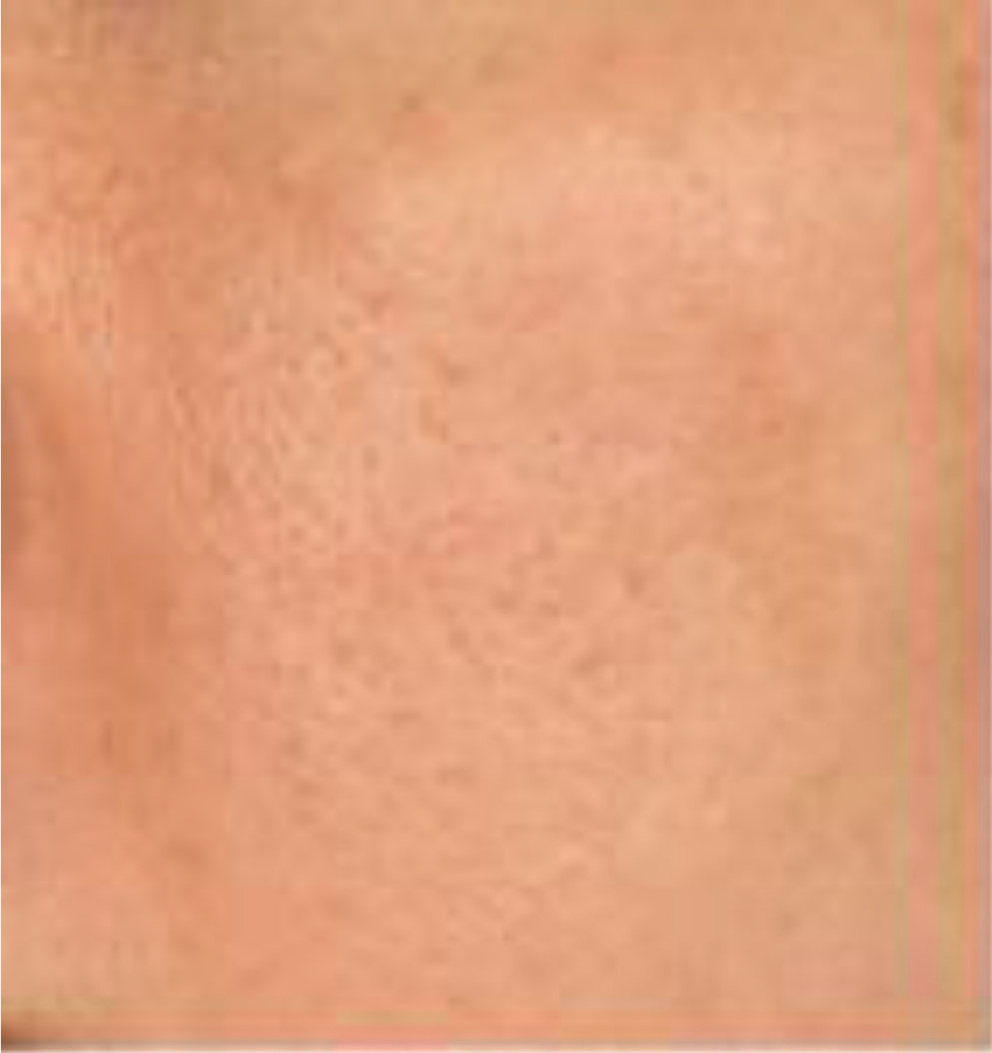
Lip surface dryness	Degree of flaking on the lips	0–4	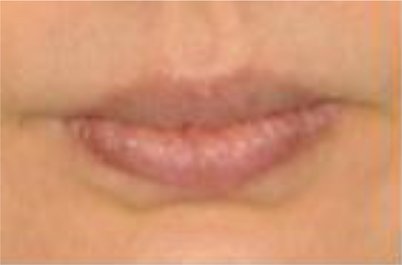
Chin sagging	Sagging severity of the lower parts on each side of the chin	0–6	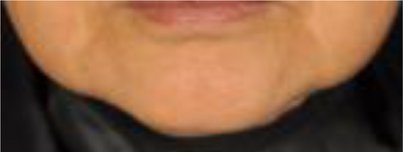

**[Visual Assessment by Dermatologists]**

Five dermatologists evaluated the severity of wrinkles, pigmentation, pores, lip dryness, and jawline sagging for each segmented facial region. Assessments were performed using the standard photographic grade from the Skin Aging Atlas of Asian Type [18], and scores were assigned on an integer scale, as shown in Table 2, with lower scores indicating a better skin condition. The final grade for each item was determined using a consensus‐based rule, in which the grade agreed upon by at least three of the five board‐certified dermatologists was assigned as the ground truth. Dermatologist grading was performed under strictly blinded conditions, without access to participant information (including age, sex, and medical history) or physiological measurement results, to minimize potential sources of evaluator bias.

**[Physiological Measurements Using Non‐Invasive Devices]**

Physiological skin parameters—including hydration (forehead, cheeks, chin), elasticity (forehead, cheeks, chin), pigmentation (full face, lesion count), wrinkle severity (Crow's feet area), and pore visibility (cheeks)—were measured under controlled environmental conditions (temperature and humidity).

Skin hydration was measured using the Corneometer CM825 (Courage & Khazaka Electronic GmbH, Germany) at four sites: the central forehead, both cheeks, and the center of the chin. Skin elasticity was evaluated at the same sites using the Cutometer MPA 580 (Courage & Khazaka Electronic GmbH, Germany). Crow's feet wrinkles on both sides of the eyes were assessed using PRIMOS Lite (GFMesstechnik GmbH, Germany). Enlarged and visible pores were measured on both cheeks, and facial pigmentation was evaluated in the frontal face using the VISIA‐CR 2.3 system (Canfield Scientific, USA).

### 2.3 Deep Neural Network Architecture

We developed two deep neural networks: a classification network to predict visual skin condition grades and a regression network to predict physiological skin parameters. Both networks employed CoAtNet‐4 [16] as the backbone architecture, which effectively combines the strengths of convolutional neural networks (CNNs) [19] and Transformer‐based models [20].

**FIGURE 2:**
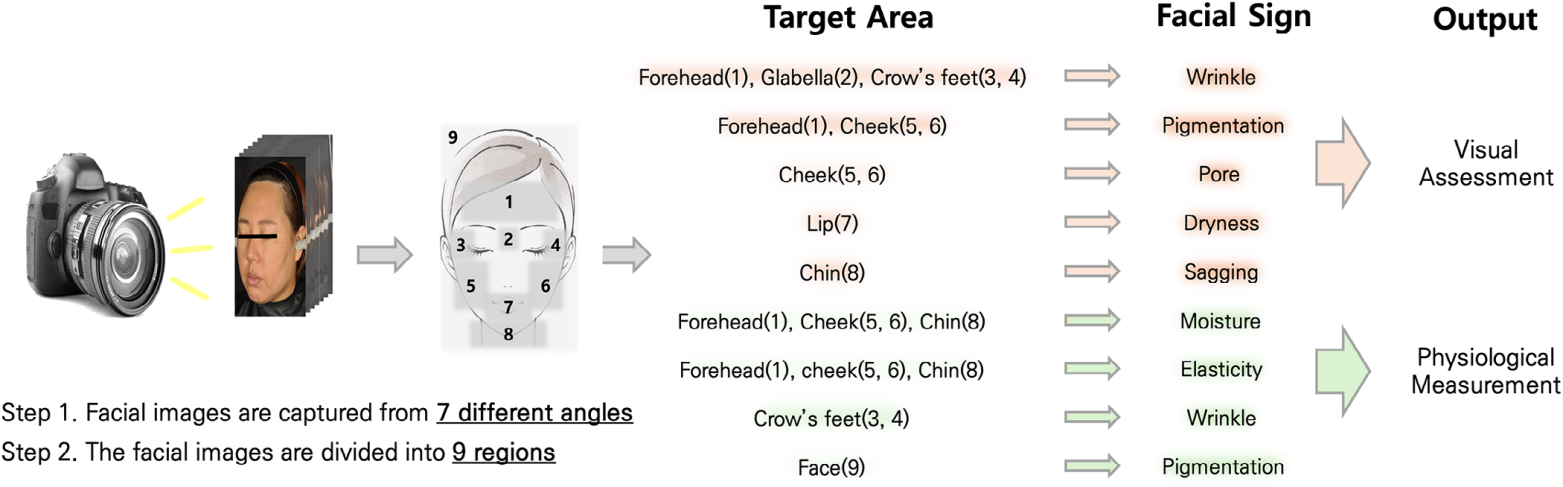
Overview of an AI‐based skin evaluation framework consisting of 9 independently trained models. Facial images are captured from seven different angles using a digital camera and segmented into key facial regions (e.g., forehead‐1, glabella‐2, eye area‐3,4, cheeks‐5,6, lips‐7, chin‐8, face‐9). Each model is assigned to a specific facial sign and task type. For wrinkle and pigmentation, both classification (grade) and regression (measurement) models are separately trained, even though they share the same facial sign. No feature extractor is shared across tasks.

CNNs, known for their ability to capture local features, are particularly effective in extracting fine‐grained skin characteristics such as texture, pores, and wrinkles. In contrast, Transformer architectures are well‐suited for modeling global contextual information and are thus advantageous for analyzing overall skin tone and the broader spatial distribution of dermatological conditions. CoAtNet leverages convolutional layers in the early stages to learn localized representations and utilizes self‐attention [15] mechanisms in the later stages to capture long‐range dependencies. This design enables the model to effectively learn both local features—such as fine texture or pore structure—and global skin condition features across broader regions, such as the cheeks and jawline. Figure 2 presents the overall framework of the CoAtNet‐based skin condition assessment system.

As shown in Table 2, visual skin condition grades were categorized into seven levels (ranging from 0 to 6). The distribution of dermatologist‐assigned visual skin grades across facial signs is summarized in Table 3. Most samples were concentrated in intermediate grades, whereas extreme grade levels (e.g., grades 0 and 6) were relatively rare in the dataset.

**TABLE 3:** Distribution of dermatologist‐assigned visual skin grades across facial signs. Values are presented as n (%). For bilateral facial regions (e.g., crow's feet wrinkles, cheek pigmentation, and cheek pores), the counts from the left and right sides were combined.

Facial Sign	Grade 0	Grade 1	Grade 2	Grade 3	Grade 4	Grade 5	Grade 6	Total
Forehead wrinkles	44 (4.10%)	391 (36.47%)	261 (24.35%)	180 (16.79%)	84 (7.84%)	63 (5.88%)	49 (4.57%)	1072 (100%)
Crow's feet wrinkles	156 (7.28%)	712 (33.21%)	320 (14.93%)	322 (15.02%)	207 (9.66%)	211 (9.84%)	216 (10.07%)	2144 (100%)
Glabellar wrinkles	225 (20.99%)	463 (43.19%)	132 (12.31%)	117 (10.91%)	35 (3.26%)	66 (6.16%)	34 (3.17%)	1072 (100%)
Forehead pigmentation	223 (20.80%)	543 (50.65%)	206 (19.22%)	88 (8.21%)	8 (0.75%)	4 (0.37%)	—	1072 (100%)
Cheek pigmentation	65 (3.03%)	601 (28.03%)	572 (26.68%)	581 (27.10%)	228 (10.63%)	97 (4.52%)	—	2144 (100%)
Cheek pores	56 (2.61%)	374 (17.44%)	1296 (60.45%)	276 (12.87%)	118 (5.50%)	24 (1.12%)	—	2144 (100%)
Lip surface dryness	23 (2.15%)	177 (16.51%)	650 (60.63%)	198 (18.47%)	24 (2.24%)	—	—	1072 (100%)
Chin sagging	497 (46.36%)	217 (20.24%)	139 (12.97%)	125 (11.66%)	56 (5.22%)	36 (3.36%)	2 (0.19%)	1072 (100%)

To provide visual examples of the grading scale used in this study, representative sample images corresponding to each grade level are presented for cheek pores in Table 4.

**TABLE 4:** Representative images of dermatologist‐assigned cheek pore grades. Each column corresponds to grades 0–5 used in this study.

Grade 0	Grade 1	Grade 2	Grade 3	Grade 4	Grade 5
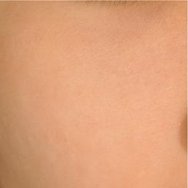	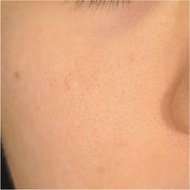	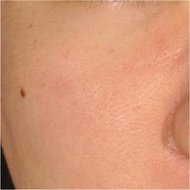	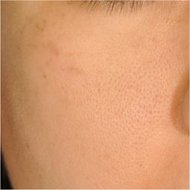	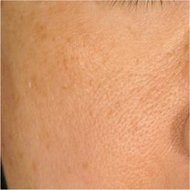	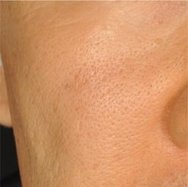

Because such imbalance is common in real‐world datasets collected from the general population, we adopted a Class‐Balanced Focal Loss that incorporates the concept of the effective number of samples per class. Specifically, the effective number [21] was defined as

$$E_{n}^{y}=\frac{1-\beta^{n_{y}}}{1-\beta }\;,$$

where $n_{y}$ is the number of samples in each class and β∈[0,1) is a hyperparameter that controls the weighting decay [22]. We set β to 0.999 in our experiments.

The final feature vector z, extracted by the CoAtNet backbone, was passed through a fully connected layer (Wz+b) followed by a softmax function to calculate the probabilities of a grade as

$$P\left(y\;=\;c|z\right)=\frac{\exp\left(W_{c}^{⊤}z+b_{c}\right)}{\sum_{j=1}^{C}\exp\left(W_{j}^{⊤}z+b_{j}\right)}\;,$$

where C is the number of grades, c∈{1,…,C} denotes the index of the grade class, and $z\in R^{1536}$ is the 1536‐dimensional feature vector extracted from the region image.

Similarly, for the prediction of physiological indicators, the regressor generated the output $\hat{y}=\;\mathrm{Wz}+b$ by applying a fully connected layer to the feature vector extracted by the backbone network, where W represents the weight matrix and b the bias vector of the regressor. Details of the backbone architecture are provided in Figure S2, and the stage‐wise spatial resolutions and channel widths of the network are summarized in Table S2.

### 2.4 Model Development and Evaluation Strategy

To evaluate the influence of image‐acquisition modality, independent models were trained using DSLR, Tablet, and Phone images. Each model was trained and evaluated primarily on images acquired from the corresponding device. To further assess generalizability across image‐acquisition conditions, the trained models were additionally evaluated on test sets acquired from other devices, and the results are summarized in Table 5.

**TABLE 5:** Cross‐device generalization performance of visual‐grade prediction models trained on DSLR images and evaluated on tablet and smartphone test sets. Values are presented as point estimates with 95% confidence intervals.

	Tablet images			Phone images		
Facial sign	%MAE = 0	%MAE ≤ 1	%MAE ≤ 2	%MAE = 0	%MAE ≤ 1	%MAE ≤ 2
Forehead wrinkles	0.38 [0.37, 0.39]	0.73 [0.71, 0.76]	0.88 [0.86, 0.89]	0.38 [0.36, 0.40]	0.73 [0.68, 0.78]	0.91 [0.85, 0.96]
Crow's feet wrinkles	0.38 [0.34, 0.41]	0.75 [0.68, 0.82]	0.89 [0.84, 0.95]	0.31 [0.27, 0.36]	0.63 [0.57, 0.69]	0.83 [0.76, 0.90]
Glabellar wrinkles	0.44 [0.40, 0.47]	0.88 [0.84, 0.91]	0.97 [0.97, 0.98]	0.34 [0.27, 0.42]	0.77 [0.73, 0.82]	0.91 [0.87, 0.95]
Forehead pigmentation	0.49 [0.35, 0.62]	0.93 [0.87, 0.98]	1.00 [0.99, 1.00]	0.48 [0.42, 0.54]	0.89 [0.84, 0.95]	0.98 [0.97, 0.99]
Cheek pigmentation	0.44 [0.39, 0.49]	0.87 [0.85, 0.90]	0.98 [0.96, 1.00]	0.39 [0.26, 0.52]	0.82 [0.73, 0.92]	0.96 [0.93, 0.99]
Cheek pores	0.48 [0.35, 0.60]	0.90 [0.85, 0.95]	0.99 [0.99, 1.00]	0.48 [0.41, 0.55]	0.89 [0.86, 0.92]	0.98 [0.97, 0.99]
Lip surface dryness	0.51 [0.38, 0.65]	0.92 [0.86, 0.98]	1.00 [0.99, 1.00]	0.48 [0.29, 0.68]	0.90 [0.83, 0.98]	0.99 [0.98, 1.00]
Chin sagging	0.30 [0.15, 0.45]	0.75 [0.71, 0.79]	0.95 [0.93, 0.97]	0.40 [0.30, 0.51]	0.83 [0.80, 0.86]	0.96 [0.94, 0.98]

To benchmark the proposed architecture against existing deep learning approaches, additional models based on ResNet50, EfficientNet‐B0 [23], and MobileNetV3‐Large [24] were trained and evaluated using the same training, validation, and test splits. All models were implemented using identical preprocessing procedures and evaluation protocols to ensure a fair comparison. The comparative performance of the proposed model and baseline architectures is summarized in Table 6.

**TABLE 6:** Comparative performance of CoAtNet‐4 and baseline architectures for visual‐grade prediction on the DSLR test set. Values are presented as point estimates with 95% confidence intervals.

Model	MAE	Pearson r	%MAE = 0	%MAE ≤ 1	%MAE ≤ 2
CoAtNet‐4	0.60 [0.58, 0.63]	0.82 [0.81, 0.83]	51.15 [48.83, 53.47]	93.26 [92.33, 94.19]	99.43 [99.16, 99.70]
ResNet50	0.63 [0.61, 0.64]	0.79 [0.77, 0.81]	50.90 [48.98, 52.81]	91.52 [90.39, 92.64]	98.86 [98.35, 99.37]
EfficientNet‐B0	0.65 [0.62, 0.69]	0.76 [0.74, 0.78]	49.50 [47.61, 51.38]	89.80 [88.91, 90.69]	98.32 [97.88, 98.77]
MobileNetV3‐Large	0.69 [0.67, 0.72]	0.72 [0.70, 0.73]	48.05 [46.09, 50.01]	87.87 [87.12, 88.61]	97.60 [96.97, 98.22]

The models were trained for 100 epochs using the Adam optimizer with an initial learning rate of 1 × 10^−^^4^. A cosine learning‐rate schedule with five warm‐up epochs and a minimum learning rate of 1 × 10^−^^6^ was employed. The batch size was set to 8 for CoAtNet‐4 and 32 for ResNet50, EfficientNet‐B0, and MobileNetV3‐Large. Classification models were optimized using class‐balanced focal loss (γ = 2), whereas regression models were trained using Huber loss.

### 2.5 Statistical Analysis

For the facial image dataset comprising 1,099 participants, train, validation, and test sets were constructed at the participant level using a 7:1:2 ratio. All images from a given participant, including multi‐angle images and cropped facial regions, were assigned exclusively to a single partition. The numbers of participants in each partition and overlap checks between partitions are summarized in Table 7. Model performance was averaged across four independent random splits.

**TABLE 7:** Participant‐level train/validation/test partitioning and overlap verification for each facial assessment task. The numbers of unique subjects assigned to the training, validation, and test sets are shown for each task and facial region. Train‐Val, Train‐Test, and Val‐Test indicate the number of overlapping subjects between the corresponding partitions. L and R denote the left and right facial regions, respectively.

Facial sign	Train subjects	Val subjects	Test subjects	Train‐Val	Train‐Test	Val‐Test
Forehead wrinkles	746	105	221	0	0	0
Crow's feet wrinkles (L)	747	106	219	0	0	0
Crow's feet wrinkles (R)	748	101	223	0	0	0
Glabellar wrinkles	747	104	221	0	0	0
Forehead pigmentation	748	104	220	0	0	0
Cheek pigmentation (L)	754	102	216	0	0	0
Cheek pigmentation (R)	741	108	223	0	0	0
Cheek pores (L)	747	104	221	0	0	0
Cheek pores (R)	748	104	220	0	0	0
Lip surface dryness	747	105	220	0	0	0
Chin sagging	746	103	221	0	0	0

To verify that the participant‐level random train–test splitting did not introduce age‐related sampling bias, we analyzed the age distribution of the test datasets across four independent experiments. As summarized in Table 8, the proportions of each age group in the test sets closely matched the original dataset composition.

**TABLE 8:** Age distribution (%) of the test sets across four independent experiments (mean ± SD).

Facial sign	14–19	20–29	30–39	40–49	50–59	60–69
Forehead wrinkles	11.31 ± 1.92	17.31 ± 2.99	17.42 ± 2.49	17.99 ± 0.77	18.21 ± 1.00	17.76 ± 2.96
Crow's feet wrinkles	9.09 ± 0.73	16.70 ± 1.48	17.02 ± 0.81	20.30 ± 0.55	17.87 ± 2.61	19.03 ± 2.06
Glabellar wrinkles	9.16 ± 2.23	18.67 ± 2.32	18.10 ± 2.06	19.23 ± 3.48	16.74 ± 3.02	18.10 ± 2.48
Forehead pigmentation	9.43 ± 1.14	17.95 ± 1.68	17.73 ± 0.91	18.52 ± 3.61	18.86 ± 1.20	17.50 ± 3.33
Cheek pigmentation	9.11 ± 0.82	16.87 ± 2.33	18.02 ± 0.61	18.54 ± 3.05	19.85 ± 1.90	17.61 ± 0.99
Cheek pores	8.91 ± 1.19	19.49 ± 1.58	17.15 ± 1.98	18.60 ± 1.73	18.71 ± 2.78	17.15 ± 1.96
Lip surface dryness	9.66 ± 0.68	18.52 ± 2.14	18.41 ± 0.26	18.18 ± 4.91	18.30 ± 3.59	16.93 ± 1.72
Chin sagging	10.52 ± 0.77	18.21 ± 2.57	17.65 ± 1.11	18.33 ± 1.50	16.97 ± 1.20	18.33 ± 1.20

Given that the human face is a three‐dimensional curved surface, visual perception of skin features, such as wrinkle depth and pigmentation, can vary due to differences in facial geometry and light reflection angles. To address these variations effectively, images captured from seven different angles were used for training. For testing, images from the frontal view and ± 30° angles were selected to consider typical clinical evaluation conditions.

For each skin assessment category, the corresponding segmented facial regions were used, as specified in Table 2. For example, in the case of wrinkle assessment, four facial regions (forehead, glabella, and both crow's feet areas) were extracted from images taken at seven angles, yielding a total of 24,640 cropped training images (880 participants × 7 angles × 4 regions). For testing, 2,620 cropped images (220 participants × 3 angles × 4 regions) were used. Table S1 in the Supplementary information summarizes the number of training and test images for each skin evaluation category.

Unlike conventional classification tasks, skin grading involves an inherent ordinal relationship among the categories. Therefore, the degree of discrepancy between the predicted and ground truth grades serves as a meaningful indicator of model performance. To capture both categorical accuracy and ordinal sensitivity, the performance of the proposed visual skin grade classification model was evaluated using accuracy and mean absolute error (MAE), with ground truth labels assigned by board‐certified dermatologists. For the prediction of physiological skin indicators, performance was assessed using the mean absolute percentage error (MAPE), the normalized mean absolute error (NMAE), and the correlation coefficient between the predicted and measured values. As the measurement ranges varied across devices, prediction errors were normalized as a proportion of the actual values.

## 3 RESULTS

### 3.1 Subject Demographics

Between July and October 2023, a total of 1,099 Korean participants (male and female, aged 14–69 years) were enrolled. As summarized in Table 1, the cohort was evenly balanced across sex and age groups, comprising 550 females and 549 males. The mean ages were 41.56±15.97 years for females and 41.46±15.99 years for males. The adolescent group (14–19 years) included 55 participants of each sex, whereas all other age decades (20s to 60s) consisted of 99 participants per sex, except for the male group in their 60s (*n* = 98).

### 3.2 Model Performance in Predicting Dermatologist Visual Grades

The CoAtNet‐based classification model demonstrated consistently high performance in predicting dermatologist‐assigned visual skin grades (Table 9). Exact grade accuracies ranged from 45.4 % to 58.7 % across all facial attributes.

**TABLE 9:** Device‐specific visual assessment prediction performance of independently trained DSLR, tablet, and smartphone models. Values are presented as point estimates with 95% confidence intervals.

Facial sign	Device	MAE	%MAE = 0	%MAE ≤ 1	%MAE ≤ 2	Pearson r
Forehead wrinkles	DSLR	0.61 [0.56, 0.66]	50.3 [46.0, 54.6]	92.8 [90.7, 94.9]	99.6 [99.2, 99.9]	0.86 [0.84, 0.88]
	Tablet	0.81 [0.77, 0.85]	41.2 [34.7, 47.8]	84.7 [83.1, 86.2]	96.5 [95.0, 97.9]	0.72 [0.69, 0.74]
	Phone	0.75 [0.68, 0.81]	42.8 [38.6, 47.0]	87.9 [82.4, 93.5]	98.4 [97.4, 99.4]	0.78 [0.75, 0.82]
Crow's feet wrinkles	DSLR	0.71 [0.65, 0.76]	45.4 [39.6, 51.2]	89.8 [88.2, 91.5]	98.8 [98.1, 99.4]	0.87 [0.86, 0.88]
	Tablet	0.75 [0.71, 0.79]	42.4 [41.6, 43.1]	88.2 [83.1, 93.3]	98.5 [97.8, 99.2]	0.85 [0.82, 0.88]
	Phone	0.82 [0.79, 0.86]	39.6 [36.1, 43.1]	84.2 [83.8, 84.5]	97.7 [97.3, 98.2]	0.83 [0.81, 0.86]
Glabellar wrinkles	DSLR	0.64 [0.60, 0.68]	49.2 [43.8, 54.7]	91.9 [90.2, 93.5]	98.6 [98.1, 99.2]	0.86 [0.84, 0.87]
	Tablet	0.70 [0.68, 0.72]	45.8 [44.0, 47.5]	89.1 [86.8, 91.4]	98.4 [97.0, 99.7]	0.83 [0.81, 0.84]
	Phone	0.83 [0.78, 0.88]	38.8 [34.6, 42.9]	85.0 [83.7, 86.4]	96.1 [94.4, 97.8]	0.76 [0.72, 0.79]
Forehead pigmentation	DSLR	0.51 [0.36, 0.65]	58.7 [43.5, 73.8]	97.0 [95.0, 99.1]	99.8 [99.5, 100.0]	0.77 [0.72, 0.82]
	Tablet	0.57 [0.50, 0.64]	55.2 [49.9, 60.5]	94.2 [89.7, 98.8]	99.4 [98.2, 100.0]	0.67 [0.58, 0.77]
	Phone	0.62 [0.57, 0.67]	50.5 [45.9, 55.1]	93.5 [89.6, 97.4]	99.2 [98.2, 100.0]	0.65 [0.61, 0.69]
Cheek pigmentation	DSLR	0.59 [0.52, 0.66]	50.5 [44.7, 56.3]	95.1 [93.2, 96.9]	99.8 [99.5, 100.0]	0.80 [0.78, 0.82]
	Tablet	0.66 [0.58, 0.74]	46.4 [40.7, 52.1]	92.3 [88.5, 96.0]	99.6 [99.2, 100.0]	0.75 [0.70, 0.79]
	Phone	0.71 [0.60, 0.83]	44.6 [35.6, 53.6]	89.2 [84.9, 93.6]	98.8 [97.4, 100.0]	0.72 [0.68, 0.75]
Cheek pores	DSLR	0.60 [0.47, 0.74]	50.8 [38.2, 63.4]	94.5 [90.6, 98.4]	99.9 [99.5, 100.0]	0.61 [0.58, 0.64]
	Tablet	0.72 [0.36, 1.08]	45.4 [28.8, 62.0]	89.0 [73.8, 100.0]	97.2 [90.2, 100.0]	0.55 [0.49, 0.61]
	Phone	0.69 [0.61, 0.77]	45.5 [35.5, 55.5]	90.6 [87.8, 93.5]	99.0 [98.3, 99.8]	0.46 [0.42, 0.51]
Lip surface dryness	DSLR	0.56 [0.47, 0.66]	55.6 [46.3, 64.9]	94.0 [92.2, 95.8]	99.6 [98.7, 100.0]	0.25 [0.12, 0.39]
	Tablet	0.58 [0.42, 0.74]	54.5 [45.6, 63.4]	92.9 [89.3, 96.5]	99.5 [98.8, 100.0]	0.20 [0.12, 0.27]
	Phone	0.66 [0.54, 0.78]	50.9 [43.5, 58.3]	91.0 [86.7, 95.2]	99.0 [97.5, 100.0]	0.12 [0.00, 0.24]
Chin sagging	DSLR	0.62 [0.50, 0.74]	51.0 [39.4, 62.6]	91.1 [89.4, 92.9]	99.4 [99.2, 99.7]	0.83 [0.80, 0.86]
	Tablet	0.65 [0.59, 0.72]	51.5 [45.9, 57.2]	88.9 [84.6, 93.2]	98.2 [97.3, 99.1]	0.79 [0.75, 0.82]
	Phone	0.65 [0.60, 0.69]	53.0 [50.1, 55.9]	88.2 [87.2, 89.1]	97.5 [96.5, 98.6]	0.77 [0.75, 0.80]

For wrinkle assessment, exact accuracies were 50.3% for forehead wrinkles, 45.4% for crow's feet, and 49.2% for glabellar wrinkles. With a tolerance of ±1 grade, accuracies increased to 92.8%, 89.8%, and 91.9%, respectively. More than 98% of predictions fell within ±2 grades of the ground truth. Correlation coefficients were strong across all wrinkle types (*r* = 0.86–0.87, all p<0.001).

Pigmentation showed the highest accuracy among all visual grading categories. Exact accuracies were 58.7% for forehead pigmentation and 50.5% for cheek pigmentation. The ±1 grade accuracies were 97.0% and 95.1%, respectively, with high correlations (forehead *r* = 0.77; cheek *r* = 0.80; both p<0.001).

For cheek pores, the exact accuracy was 50.8%, with 94.5% within ±1 grade and 99.9% within ±2 grades (*r* = 0.61, p<0.001). Chin sagging achieved 51.0% accuracy, 91.1% within ±1 grade, and 99.4% within ±2 grades, with a strong correlation (*r* = 0.83, p<0.001). Lip surface dryness achieved 55.6% accuracy, with 94.0% within ±1 grade and 99.6% within ±2 grades. Although the correlation was lower (*r* = 0.25), it remained statistically significant (p<0.001).

To further investigate whether the observed performance could be influenced by the grading range, we analyzed the mean absolute error (MAE) across grade levels for each facial sign. As summarized in Table S7, the MAE values remained generally below 1 across most grade levels and facial signs. Although prediction errors may appear smaller when the grading range is narrow, these results suggest that the model performance cannot be explained solely by the grading range.

### 3.3 Physiological Measurement Prediction Performance

The CoAtNet‐based regression model demonstrated varying degrees of correlation with physiological skin measurements obtained through non‐invasive devices (Table 10). Hydration showed moderate correlations across all sites (forehead *r* = 0.33; cheek *r* = 0.38; chin *r* = 0.50; all p<0.001). Elasticity demonstrated region‐specific correlations, with the cheek showing the strongest association (*r* = 0.60), followed by the forehead (*r* = 0.48) and chin (*r* = 0.26). Crow's feet wrinkle depth measured by PRIMOS Lite correlated well with AI predictions (*r* = 0.70; p<0.001). Cheek pore visibility showed a robust correlation (*r* = 0.79; p<0.001). Pigmentation spot count exhibited the strongest correlation among all physiological indicators (*r* = 0.92; p<0.001), indicating excellent predictive capability.

**TABLE 10:** Device‐specific physiological measurement prediction performance. Mean absolute error (MAE) and Pearson correlation coefficients are reported for each physiological parameter.

Facial sign	DSLR		Tablet		Phone	
	MAE	Pearson r	MAE	Pearson r	MAE	Pearson r
Crow's feet wrinkles	0.0931	0.7041	0.0992	0.6610	0.1042	0.6233
Front‐face pigmentation spots	0.0704	0.9161	0.0830	0.8873	0.0814	0.8805
Cheeks pore visibility	0.0966	0.7924	0.1040	0.7481	0.1392	0.5131
Forehead elasticity	0.1355	0.4791	0.1472	0.2616	0.1463	0.2160
Cheek elasticity	0.1214	0.5992	0.1289	0.5658	0.1366	0.4662
Chin elasticity	0.1552	0.2582	0.1630	0.1969	0.1581	0.1241
Forehead hydration	0.1476	0.3265	0.1452	0.3073	0.1501	0.2479
Cheek hydration	0.1264	0.3809	0.1341	0.2607	0.1330	0.2938
Chin hydration	0.1329	0.4963	0.1377	0.4984	0.1273	0.4724

**FIGURE 3:**
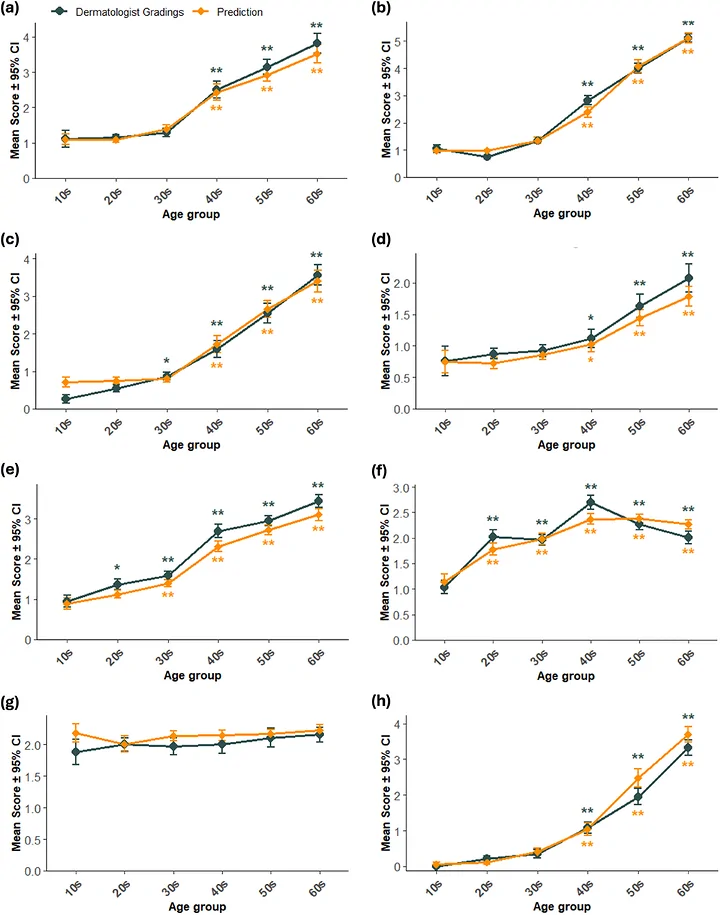
Bland–Altman plots comparing AI‐predicted values and device‐based physiological measurements for hydration, elasticity, wrinkle severity, pore visibility, and pigmentation spot count. The solid line indicates the mean difference, and the dashed lines represent the 95% limits of agreement.

To further evaluate agreement between AI‐predicted values and device‐based measurements, Bland–Altman analyses were performed for all physiological parameters (Figure 3). Most observations fell within the 95% limits of agreement, with no substantial systematic bias observed across the measurement range.

### 3.4 Comparison With Existing Deep Learning Architectures

Table 6 compares the proposed hybrid Transformer–CNN architecture with widely used CNN‐based models, including ResNet50, EfficientNet‐B0, and MobileNetV3‐Large. Across most facial signs, the proposed model achieved superior prediction performance, yielding lower MAE values and higher Pearson correlation coefficients. These results suggest that integrating Transformer‐based global contextual modeling with CNN‐based local feature extraction provides a more effective representation for facial skin assessment than conventional CNN architectures.

### 3.5 Age‐related Trends and Agreement With Dermatologists

**FIGURE 4:**
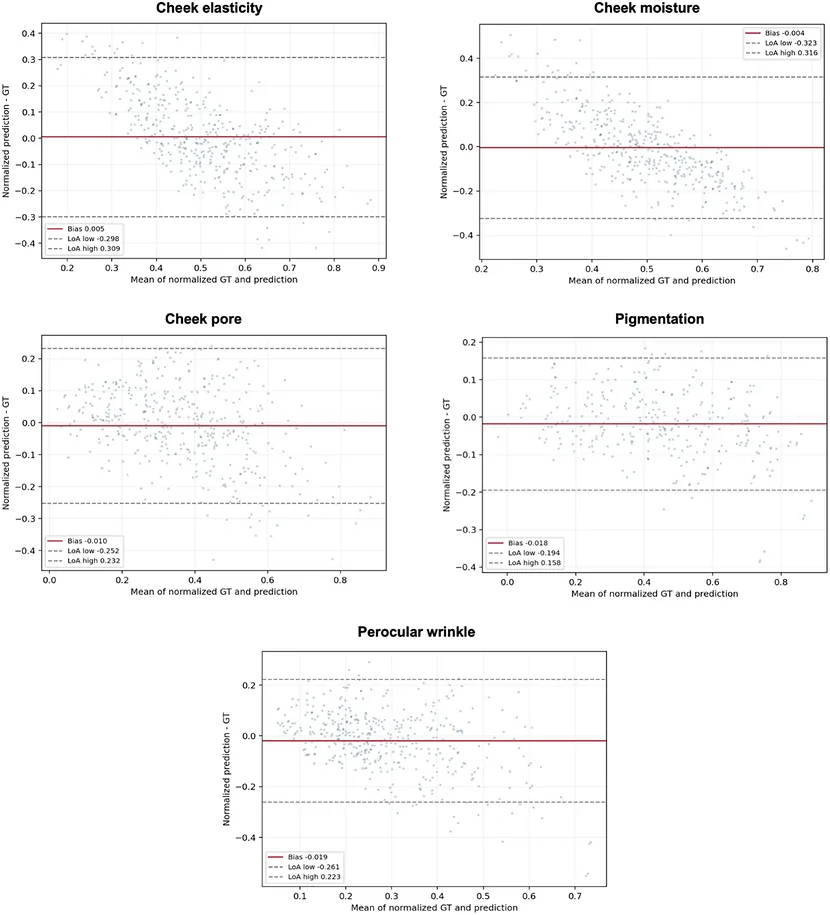
a‐h, Mean ± CI for visual scores according to age. (a) Wrinkles of forehead, (b) Wrinkles of crow's feet, (c) Wrinkles of glabellar forehead, (d) Pigmentation of forehead, (e) Pigmentation of cheek, (f) Pores of cheeks, (g) Dryness of lips, (h) Sagging of chin. Filled circles (●) represent Dermatologist Gradings and filled diamonds (◆) represent Prediction values. Asterisks indicate statistically significant differences compared to the 10s age group based on one‐way ANOVA with Tukey HSD post‐hoc test (* *p* < 0.05, ** *p* < 0.001). Significance symbols above data points refer to Dermatologist Gradings; symbols below data points refer to Prediction values.

Figure 4(a–h) illustrates age‐dependent changes in visual skin scores based on dermatologist assessments and AI predictions. For most facial features, such as forehead, crow's feet, and glabellar wrinkles; forehead and cheek pigmentation; cheek pores; and chin sagging, the AI model accurately reproduced the age‐related progression observed by dermatologists. Both assessments demonstrated accelerated worsening of these aging signs among individuals in their 40s, consistent with established clinical patterns.

In contrast, lip surface dryness showed no clear age‐related trend, suggesting that more substantial interindividual variability, likely influenced by personal physiological or behavioral factors, was more pronounced.

### 3.6 Impact of Image Source (DSLR vs. Mobile Devices)

Model performance remained robust when evaluated on tablet and smartphone images, although accuracy was lower than on high‐resolution DSLR images (Table 9). Across most facial signs, ≤1 grade accuracies exceeded 84.2% for both mobile devices, indicating that clinically meaningful assessments are feasible using consumer‐grade images.

Cheek pores exhibited the highest ≤1 grade accuracies (Tablet:94.2%; Phone: 93.5%). However, exact grade accuracies (%MAE = 0) were consistently lower for mobile images. For example, exact accuracy for forehead pigmentation decreased from 58.7% (DSLR) to 55.2% (Tablet) and 50.5% (Phone), highlighting the sensitivity of fine‐grade prediction to image resolution and quality.

Similarly, performance in predicting physiological measurements decreased when using mobile images. While pigmentation spot count maintained strong correlations for both Tablet and Phone images (*r* = 0.89 and 0.88, respectively), other attributes showed substantial reductions. Cheek pore visibility declined from *r* = 0.79 (DSLR) to *r* = 0.51 (Phone), and chin hydration decreased from *r* = 0.50 to *r* = 0.47. Nevertheless, all correlations remained statistically significant (p<0.001).

To evaluate robustness across image‐acquisition conditions, DSLR‐trained models were additionally tested on tablet and smartphone images (Table 5). Although performance decreased compared with device‐matched evaluation, most facial signs maintained high ≤1 grade accuracies, demonstrating reasonable cross‐device generalizability. Forehead pigmentation showed the strongest cross‐device performance, whereas crow's feet wrinkles exhibited the largest performance degradation on smartphone images.

### 3.7 Model Interpretability Using Grad‐CAM

**FIGURE 5:**
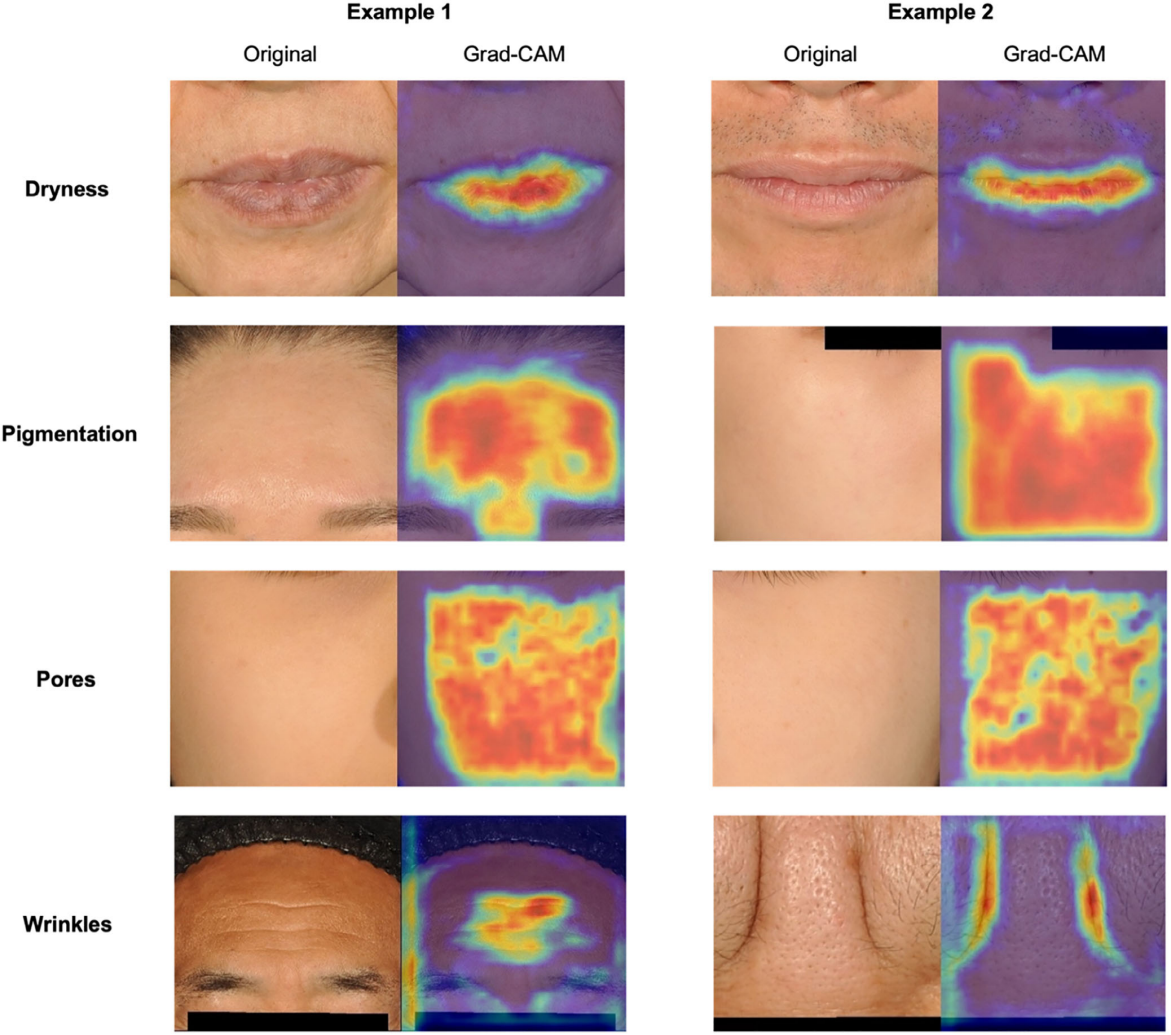
Representative Grad‐CAM visualizations for visual skin assessment prediction using independent test‐set images. Original cropped facial‐region images and corresponding Grad‐CAM overlays are shown for dryness, pigmentation, pores, and wrinkles. Warmer colors indicate regions with greater contribution to the model output.

Representative Grad‐CAM visualizations are presented in Figure 5. The model consistently focused on clinically relevant facial regions associated with each assessment category, including wrinkle lines, pigmented lesions, pore‐rich areas, and dry lip regions. These visualizations indicate that the model's predictions were primarily driven by dermatologically meaningful image features.

## 4 Discussion

In this study, we developed an AI‐based skin assessment framework using CoAtNet, a hybrid deep learning architecture that integrates the strengths of convolutional neural networks and Transformers. While recent advances in dermatological AI have predominantly relied on traditional CNN architectures to evaluate localized skin lesions or perform single‐task classifications [8, 13, 14], the proposed model demonstrated the capability to simultaneously predict dermatologist‐assigned visual grades and objective physiological measurements using only multi‐angle facial images, thereby extending the capabilities of conventional AI skin‐assessment systems that primarily focus on visual grading [11, 25, 26].

### 4.1 Comparison with Previous AI‐Based Skin Assessment Studies

The majority of prior AI‐based skin assessment systems have focused predominantly on appearance‐based classification, encompassing tasks such as wrinkle severity grading, pigmentation mapping, acne severity scoring, and lesion recognition derived from facial photographs [9, 10, 11, 12, 13, 14, 27, 28]. Although these approaches have demonstrated the feasibility of automated visual skin analysis, they remain largely constrained to surface‐level grading tasks and do not typically extend to the estimation of quantitative physiological skin parameters. This distinction carries clinical relevance, as skin aging and overall skin condition are inherently multidimensional phenomena encompassing both visible morphological changes and less overt biophysical properties [1, 2, 3, 6].

### 4.2 Clinical Implications of Visual Grading Prediction

The model exhibited strong performance across a range of visual aging markers, achieving exact‐grade accuracies of 45.4–58.7% and exceeding 92% accuracy for most attributes when ±1‐grade tolerance was applied. Given the inherent subjectivity and inter‐rater variability in clinical visual grading [4, 5], these results indicate that the AI model can reliably approximate expert assessments. Moreover, the model successfully reproduced age‐related progression patterns [1, 2, 3]—including the characteristic acceleration of wrinkles, pigmentation, and sagging in individuals in their 40s (Figure 4(a–h))—closely mirroring dermatologist evaluations. Compared to previous automated grading systems [10], this suggests that the model can sensitively capture biologically relevant cutaneous aging trajectories and may serve as a valuable tool for epidemiological and longitudinal research.

### 4.3 Predicting Physiological Skin Measurements

A notable strength of this work lies in its ability to predict objective physiological parameters in addition to visual grades. High correlations were observed for pigmentation spot count (*r* = 0.91), pore visibility (*r* = 0.79), and wrinkle severity (*r* = 0.70), demonstrating that the proposed model can approximate device‐based quantitative measurements using standard facial photographs. In contrast, hydration and elasticity—attributes reflecting deeper dermal characteristics—showed only moderate correlations (*r* = 0.24–0.61). Previous studies utilizing biophysical properties have noted the difficulty of correlating in vivo physiological measurements directly with 2D photographic features [6, 29]. These findings highlight the intrinsic limitations of RGB image‐based prediction and underscore the potential benefits of incorporating non‐visual or multimodal information, such as spectral, thermal, or biomechanical data, in future models.

The observed variations in correlation between AI‐predicted visual grades and physiological measurements underscore that skin assessment is multifaceted. Visual assessment reflects the overall clinical phenotype—the integrated appearance of skin—whereas physiological measurements quantify localized functional parameters. Rather than viewing one as a replacement for the other, our findings suggest that these two modalities are complementary tools; our AI framework successfully objectifies professional visual grading while providing additional insights into biophysical properties.

### 4.4 Influence of Image Quality and Real‐World Applicability

Translating AI models from controlled clinical settings to consumer environments remains a significant challenge [9]. Image quality emerged as a critical factor influencing model performance. Predictions based on mobile device images consistently showed lower accuracy than those based on high‐resolution DSLR images (e.g., forehead pigmentation: 58.7% vs 50.5%). Differences in resolution, lighting, and pose likely contributed to this decline, underscoring the importance of robustness in uncontrolled consumer environments. To address this limitation, future work should incorporate super‐resolution algorithms, unsupervised domain adaptation, and illumination‐ or pose‐invariant feature extraction techniques. These improvements may help bridge the performance gap between controlled clinical imaging and real‐world consumer‐captured images.

### 4.5 Limitations and Future Directions

The dataset consisted exclusively of Korean participants recruited from a single center, which may limit the generalizability of the proposed framework to other ethnic groups and clinical settings. Future work should prioritize external validation across ethnically diverse populations, multiple institutions, and varied image acquisition conditions to establish the broader applicability of the model.

Additionally, formal inter‐rater agreement statistics, such as Cohen's kappa or intraclass correlation coefficients, were not computed in this study, as the individual per‐rater grade records are no longer readily accessible. Future work should prospectively record and report such metrics to provide a more rigorous characterization of ground truth reliability.

A prospective validation cohort or a permanently held‐out external test set was not available in this study, which represents an important limitation. Future work should incorporate such validation strategies to provide a more definitive assessment of model generalizability [13].

## 5 Conclusion

This study presents a CoAtNet‐based deep learning framework capable of predicting both visual aging grades and physiological skin parameters from multi‐angle facial images. The model demonstrated high predictive accuracy for key aging markers and strong correlations with device‐based quantitative measurements, suggesting that clinically meaningful assessments can be achieved using standard facial photographs.

The ability to replicate age‐related trends further supports its potential use in dermatological research and long‐term monitoring of skin aging. Although performance declined when using mobile device images, clinically acceptable accuracy was maintained, supporting feasibility for future consumer‐oriented applications.

Our model provides a robust foundation for standardizing clinical visual evaluation across a wide adult age range, despite the practical limitations in adolescent sampling. By simultaneously capturing both visible aging markers and underlying physiological parameters, this framework offers a comprehensive and multifaceted approach to skin analysis, bridging the gap between subjective expert grading and objective instrumental measurement.

To enhance generalizability and practical deployment, future research should incorporate diverse ethnic populations, image‐enhancement methods, and multimodal learning strategies. Overall, the proposed AI framework shows significant promise as a foundation for next‐generation digital dermatology and personalized skincare technologies.

## Ethics Statement

The Study Protocol Was Approved By the Institutional Review Board of IEC Korea (Approval No.: IECK(1)‐IRB‐202307‐102‐06). The study Was Conducted in Accordance With the Declaration of Helsinki and ICH‐GCP Guidelines, and written informed consent was obtained from all participants prior to enrollment. For minors (participants aged 14–17 years), written informed consent was obtained from both the participants and their parents or legal guardians.

## Consent for Publication of Images

The individual appearing in Figure 1 provided written informed consent for the use and publication of their facial image.

## Conflicts of Interest

The authors declare no conflicts of interest.

## Supporting information



Supporting File 1

Supporting File 2

Supporting File 3

Supporting File 4

## Data Availability

The data that support the findings of this study are openly available in AI‐Hub at https://www.aihub.or.kr, reference number 71645.
