# Supplementary material for: Artificial Intelligence Based Skin Analysis Models for Predicting Visual Grades and Device Measured Physiological Values From Facial Images
Source: Skin Res Technol. 2026 Aug 31;32(9):e70375. doi: 10.1111/srt.70375 (PMC13530381; doi:10.1111/srt.70375)
Supplement: Supplementary file 1 [file SRT-32-e70375-s004.docx]

# Image pre-processing for model input

## Rationale for Pre-processing

Deep learning-based analysis requires all input images to share a consistent spatial resolution and aspect ratio. However, the facial-region crops extracted in this study were rectangular, and certain regions, such as the forehead and crow’s feet, displayed notably elongated aspect ratios. Directly resizing these rectangular regions to square inputs would distort anatomical features (e.g., flattening wrinkles or altering pore geometry), potentially degrading diagnostically relevant texture information. To preserve morphological fidelity, a dedicated pre-processing strategy was implemented.

## Bounding Box-Based Square Cropping

To avoid geometric distortion, we employed a bounding box–based square cropping method. For each facial region, the longer edge of the bounding box was taken as the side length of a square crop, and the crop was centered on the targeted anatomical area. This approach preserves the region’s natural proportions while producing square images compatible with the CoAtNet backbone. An illustration of the procedure is provided in Supplementary Figure S1.

## Summary of the Pre-processing Pipeline

The complete pre-processing workflow consisted of: (1) facial-landmark–based region localization, (2) bounding box extraction, (3) square cropping, (4) resizing to $256\times256$ pixels, and (5) normalization. This pipeline ensured that each model input preserved anatomical geometry and met the network’s dimensional requirements.

# Training and Test Set Construction

## Stratified Splitting at the Participant Level

Dermatologist-assigned visual grades and device-based physiological measurements exhibited uneven value distributions, with many samples concentrated within specific ranges. A simple random split at the image level could therefore lead to particular grades or measurement intervals being under-represented in the training set or disproportionately represented in the test set. Such an imbalance can bias the results and reduce performance stability.

To mitigate these issues, we performed a stratified split at the participant level. Within each grade or measurement range, 80% of participants were assigned to the training set and 20% to the test set. This method ensures that both sets reflect the original distribution and provides a more reliable basis for model development.

## Managing Multi-View Images per Participant

Because up to seven images of the same facial region were acquired at different angles, all region images belonging to a given participant were placed in the same dataset subset. This prevents information leakage caused by highly correlated multi-angle images of the same subject appearing in both the training and test sets.

## Final Dataset

The final number of regional images per facial sign, acquisition angle, and dataset split is summarized in Supplementary Table S1.

# Network Configuration for Skin Analysis

## Overview of the Framework

The proposed AI framework consists of two main components: (1) a shared backbone network for feature extraction, and (2) task-specific prediction modules for visual grading (classification) and physiological measurement (regression). The backbone is shared across tasks, while the prediction heads differ according to the target output.

## Backbone Module

We employed CoAtNet-4, a hybrid architecture that combines the strengths of convolutional neural networks (CNNs) and Transformer-based attention mechanisms. CNN blocks provide strong local feature extraction capabilities for fine textures such as pores and wrinkles, while self-attention enables integration of broader contextual information across facial structures.

The network’s stages are constructed as follows:

- **S0–S2:** MBConv-based depth-wise convolution layers [1] that capture fine-grained local structure.
- **S3–S4:** Self-attention layers [2] that model long-range dependencies and global facial context.
- **Residual connections:** Used throughout to stabilize training and preserve low-level information.

Spatial resolutions and channel widths across stages are in Supplementary Table S2. A global pooling operation applied to the S4 output yields a 1,536-dimensional embedding vector.

## Prediction Module

The 1,536-dimensional feature vector is passed to a multilayer perceptron (MLP) composed of three fully connected (FC) layers with GELU activations inserted between them (FC $\to$ GELU $\to$ FC $\to$ GELU $\to$ FC). The first and second FC layers produce 512 and 128 units, respectively.

- **Visual grading (classification):** The output dimension corresponds to the number of discrete grades for the facial sign (e.g., five outputs for a 0–4 scale).
- **Physiological measurements (regression):** The module produces a single scalar representing the predicted device value.

# Experimental Details

## Training Procedure

Models were trained for 100 epochs with a batch size of 8, using input images resized to $256\times256$ pixels. A total of nine independent models were trained: five classifiers (one per facial sign) and four regressors (wrinkle severity, pigmentation spots, hydration, and elasticity).

## Optimization and Loss Functions

**[Classification Tasks]**

- Optimizer: Adam (learning rate = 0.0001)
- Training loss: class-balanced focal loss
- Validation loss: cross-entropy loss

**[Regression Tasks]**

- Optimizer: Adam (learning rate = 0.001)
- Training loss: Smooth L1 loss
- Validation loss: L1 loss

## Computing Environment

All experiments were conducted on a server running Ubuntu 22.04, equipped with an Intel i9-10980XE CPU, 128 GB RAM, and an NVIDIA RTX 3090 GPU. Models were implemented using Python 3.8.19 and PyTorch 2.7.0.

# Ablation Study

To evaluate the contribution of specific design components, we conducted ablation studies examining the effects of: (1) the number of acquisition angles, (2) loss functions, (3) image pre-processing strategies, and (4) input modalities (grayscale vs. RGB). All other hyperparameters were kept constant for fair comparisons.

Each configuration was trained and evaluated four times using independent participant-level splits. Reported results represent the mean across the four runs. Models trained with 1, 3, and 7 camera angles were compared. Results are presented in Supplementary Table S3. Performance differences among focal loss, class-balanced focal loss, and cross-entropy loss are summarized in Supplementary Table S4. The influence of bounding box cropping versus alternative resizing methods is shown in Supplementary Table S5. Performance using grayscale, histogram-equalized grayscale, and RGB inputs is detailed in Supplementary Table S6.

## Evaluation Metrics

For classification tasks, we report mean absolute error (MAE), exact match rate (%MAE = 0), and the percentages of predictions within $\pm$1 and $\pm$2 grades of the dermatologist reference. For regression tasks, performance was assessed using Pearson correlation coefficients and MAE-based error metrics.

# References

1. Tan M, Le Q. EfficientNet: Rethinking model scaling for convolutional neural networks. Int Conf Mach Learn 2019:6105–6114.

2. Vaswani A, Shazeer N, Parmar N, et al. Attention is all you need. Adv Neural Inf Process Syst 2017;30.

3. Lin TY, Goyal P, Girshick R, He K, Dollár P. Focal loss for dense object detection. Proc IEEE Int Conf Comput Vis 2017:2980–2988.

4. Cui Y, Jia M, Lin TY, Song Y, Belongie S. Class-balanced loss based on effective number of samples. Proc IEEE/CVF Conf Comput Vis Pattern Recognit 2019:9268–9277.

# Tables

**Table S1**. Number of regional facial images available for each facial sign and acquisition angle after stratified train–test partitioning. F, frontal; L15/L30, left 15°/30° views; R15/R30, right 15°/30° views; Ft, frontal tilt; Fb, frontal bottom view.

| **Facial sign and region** | **Set** | **F** | **L15** | **L30** | **R15** | **R30** | **Ft** | **Fb** | **Total** |
| --- | --- | --- | --- | --- | --- | --- | --- | --- | --- |
| Forehead wrinkles | Train | 746 | 746 | 746 | 746 | 746 | 746 | 746 | **5,222** |
|  | Val | 105 | 105 | 105 | 105 | 105 | 105 | 105 | **735** |
|  | Test | 221 | 0 | 221 | 0 | 221 | 0 | 0 | **663** |
| Crow’s feet wrinkles (L) | Train | 748 | 747 | 748 | 0 | 0 | 748 | 748 | **3,739** |
|  | Val | 101 | 101 | 101 | 0 | 0 | 101 | 101 | **505** |
|  | Test | 223 | 0 | 223 | 0 | 0 | 0 | 0 | **446** |
| Crow’s feet wrinkles (R) | Train | 747 | 0 | 0 | 747 | 747 | 747 | 747 | **3,735** |
|  | Val | 106 | 0 | 0 | 106 | 106 | 106 | 106 | **530** |
|  | Test | 219 | 0 | 0 | 0 | 219 | 0 | 0 | **438** |
| Glabellar wrinkles | Train | 747 | 747 | 747 | 747 | 747 | 747 | 747 | **5,229** |
|  | Val | 104 | 104 | 104 | 104 | 104 | 104 | 104 | **728** |
|  | Test | 221 | 0 | 221 | 0 | 221 | 0 | 0 | **663** |
| Forehead pigmentation | Train | 748 | 748 | 748 | 748 | 748 | 748 | 748 | **5,236** |
|  | Val | 104 | 104 | 104 | 104 | 104 | 104 | 104 | **728** |
|  | Test | 220 | 0 | 220 | 0 | 220 | 0 | 0 | **660** |
| Cheek pigmentation (L) | Train | 741 | 741 | 741 | 0 | 0 | 741 | 741 | **3,705** |
|  | Val | 108 | 108 | 108 | 0 | 0 | 108 | 108 | **540** |
|  | Test | 223 | 0 | 223 | 0 | 0 | 0 | 0 | **446** |
| Cheek pigmentation (R) | Train | 754 | 0 | 0 | 754 | 754 | 754 | 754 | **3,770** |
|  | Val | 102 | 0 | 0 | 102 | 102 | 102 | 102 | **510** |
|  | Test | 216 | 0 | 0 | 0 | 216 | 0 | 0 | **432** |
| Cheek pores (L) | Train | 748 | 748 | 748 | 0 | 0 | 748 | 748 | **3,740** |
|  | Val | 104 | 104 | 104 | 0 | 0 | 104 | 104 | **520** |
|  | Test | 220 | 0 | 220 | 0 | 0 | 0 | 0 | **440** |
| Cheek pores (R) | Train | 747 | 0 | 0 | 747 | 747 | 747 | 747 | **3,735** |
|  | Val | 104 | 0 | 0 | 104 | 104 | 104 | 104 | **520** |
|  | Test | 221 | 0 | 0 | 0 | 221 | 0 | 0 | **442** |
| Lip surface dryness (lip) | Train | 747 | 747 | 747 | 747 | 747 | 747 | 747 | **5,229** |
|  | Val | 105 | 105 | 105 | 105 | 105 | 105 | 105 | **735** |
|  | Test | 220 | 0 | 220 | 0 | 220 | 0 | 0 | **660** |
| Chin sagging | Train | 746 | 746 | 746 | 746 | 746 | 0 | 0 | **3,730** |
|  | Val | 103 | 103 | 103 | 103 | 103 | 0 | 0 | **515** |
|  | Test | 221 | 0 | 221 | 0 | 221 | 0 | 0 | **663** |

**Table S2**. Spatial resolutions and channel widths across stages.

| **Stage** | **Resolution** | **Channels** |
| --- | --- | --- |
| S0 | $128\times128$ | 192 |
| S1 | $64\times64$ | 192 |
| S2 | $32\times32$ | 384 |
| S3 | $16\times16$ | 768 |
| S4 | $8\times8$ | 1,536 |

**Table S3**. Comparison of facial-sign grade classification performance according to the number of camera angles used for training. In the 1-angle configuration, only the frontal view was used; in the 3-angle configuration, frontal, left 30°, and right 30° views were used. All models were evaluated using the same three views (frontal, left 30°, right 30°). † indicates the configuration used in the main manuscript.

| **Condition** | **Facial sign** | **Pearson *r*** | **P-value** | **MAE** | **%MAE=0** | **%MAE**$\boldsymbol{\leq}$**1** | **%MAE**$\boldsymbol{\leq}$**2** |
| --- | --- | --- | --- | --- | --- | --- | --- |
| 1-angle | Forehead wrinkles | 0.70 | $<$0.0001 | 0.76 | 45.10 | 85.22 | 94.87 |
|  | Crow’s feet wrinkles | 0.81 | $<$0.0001 | 0.85 | 39.59 | 80.66 | 95.54 |
|  | Glabellar wrinkles | 0.77 | $<$0.0001 | 0.79 | 43.89 | 83.26 | 95.78 |
|  | Forehead pigmentation | 0.68 | $<$0.0001 | 0.50 | 58.18 | 92.73 | 99.39 |
|  | Cheek pigmentation | 0.69 | $<$0.0001 | 0.63 | 48.08 | 89.37 | 99.55 |
|  | Cheek pores | 0.40 | $<$0.0001 | 0.64 | 48.86 | 88.70 | 98.17 |
|  | Lip surface dryness | 0.20 | $<$0.0001 | 0.56 | 52.88 | 91.97 | 99.09 |
|  | Chin sagging | 0.47 | $<$0.0001 | 0.92 | 48.11 | 76.32 | 87.93 |
| 3-angle | Forehead wrinkles | 0.75 | $<$0.0001 | 0.73 | 41.33 | 88.08 | 97.59 |
|  | Crow’s feet wrinkles | 0.81 | $<$0.0001 | 0.82 | 40.39 | 83.18 | 94.85 |
|  | Glabellar wrinkles | 0.80 | $<$0.0001 | 0.69 | 46.30 | 88.84 | 96.23 |
|  | Forehead pigmentation | 0.70 | $<$0.0001 | 0.44 | 61.36 | 94.70 | 99.55 |
|  | Cheek pigmentation | 0.75 | $<$0.0001 | 0.59 | 49.89 | 91.74 | **99.66** |
|  | Cheek pores | 0.41 | $<$0.0001 | **0.46** | **62.79** | 92.24 | 98.63 |
|  | Lip surface dryness | 0.29 | $<$0.0001 | 0.48 | 57.42 | **95.30** | 99.70 |
|  | Chin sagging | 0.61 | $<$0.0001 | 0.79 | 50.38 | 79.94 | 93.06 |
| 7-angle^†^ | Forehead wrinkles | **0.84** | $<$0.0001 | **0.52** | **55.81** | **92.91** | **99.10** |
|  | Crow’s feet wrinkles | **0.86** | $<$0.0001 | **0.62** | **52.63** | **87.76** | **98.05** |
|  | Glabellar wrinkles | **0.85** | $<$0.0001 | **0.55** | **54.60** | **92.01** | **99.10** |
|  | Forehead pigmentation | **0.79** | $<$0.0001 | **0.33** | **69.70** | **97.58** | **99.85** |
|  | Cheek pigmentation | **0.81** | $<$0.0001 | **0.45** | **60.41** | **94.68** | **99.66** |
|  | Cheek pores | **0.57** | $<$0.0001 | 0.48 | 57.99 | **94.63** | **99.77** |
|  | Lip surface dryness | **0.36** | $<$0.0001 | **0.45** | **60.61** | 94.39 | **99.85** |
|  | Chin sagging | **0.83** | $<$0.0001 | **0.53** | **60.33** | **90.05** | **97.29** |

**Table S4**. Comparison of facial-sign grade classification performance using different loss functions (reformatted). † indicates the configuration used in the main manuscript.

| Condition | **Facial sign** | | **Pearson *r*** | **P-value** | **MAE** | **%MAE=0** | **%MAE**$\boldsymbol{\leq}$**1** | **%MAE**$\boldsymbol{\leq}$**2** |
| --- | --- | --- | --- | --- | --- | --- | --- | --- |
| CE Loss | | Forehead wrinkles | 0.73 | <0.0001 | 0.71 | 47.96 | 85.22 | 96.53 |
|  |  | Crow’s feet wrinkles | 0.79 | <0.0001 | 0.84 | 40.05 | 80.55 | 95.77 |
|  |  | Glabellar wrinkles | 0.78 | <0.0001 | 0.70 | 46.76 | 87.78 | 95.92 |
|  |  | Forehead pigmentation | 0.67 | <0.0001 | 0.43 | 63.03 | 95.45 | 98.94 |
|  |  | Cheek pigmentation | 0.76 | <0.0001 | 0.59 | 48.87 | 92.65 | **99.66** |
|  |  | Cheek pores | 0.39 | <0.0001 | 0.54 | 57.65 | 89.16 | 99.32 |
|  |  | Lip surface dryness | 0.27 | <0.0001 | 0.53 | 53.33 | 94.39 | 99.39 |
|  |  | Chin sagging | 0.74 | <0.0001 | 0.64 | 51.73 | 87.18 | 98.04 |
| Focal Loss [3] | | Forehead wrinkles | 0.80 | <0.0001 | 0.62 | 48.87 | 90.65 | 98.94 |
|  |  | Crow’s feet wrinkles | 0.84 | <0.0001 | 0.73 | 39.93 | **88.79** | **98.51** |
|  |  | Glabellar wrinkles | 0.82 | <0.0001 | 0.62 | 47.81 | 91.25 | 98.94 |
|  |  | Forehead pigmentation | 0.68 | <0.0001 | 0.47 | 58.94 | 95.61 | 98.94 |
|  |  | Cheek pigmentation | 0.74 | <0.0001 | 0.60 | 48.76 | 92.19 | **99.66** |
|  |  | Cheek pores | 0.35 | <0.0001 | 0.51 | **60.96** | 90.30 | 97.95 |
|  |  | Lip surface dryness | 0.25 | <0.0001 | 0.50 | 55.91 | **94.70** | 99.70 |
|  |  | Chin sagging | 0.79 | <0.0001 | 0.55 | 56.71 | 89.29 | **98.94** |
| CB Focal Loss^†^ [4] | | Forehead wrinkles | **0.84** | <0.0001 | **0.52** | **55.81** | **92.91** | **99.10** |
|  |  | Crow’s feet wrinkles | **0.86** | <0.0001 | **0.62** | **52.63** | 87.76 | 98.05 |
|  |  | Glabellar wrinkles | **0.85** | <0.0001 | **0.55** | **54.60** | **92.01** | **99.10** |
|  |  | Forehead pigmentation | **0.79** | <0.0001 | **0.33** | **69.70** | **97.58** | **99.85** |
|  |  | Cheek pigmentation | **0.81** | <0.0001 | **0.45** | **60.41** | **94.68** | **99.66** |
|  |  | Cheek pores | **0.57** | <0.0001 | **0.48** | 57.99 | **94.63** | **99.77** |
|  |  | Lip surface dryness | **0.36** | <0.0001 | **0.45** | **60.61** | 94.39 | **99.85** |
|  |  | Chin sagging | **0.83** | <0.0001 | **0.53** | **60.33** | **90.05** | 97.29 |

**Table S5**. Comparison of facial-sign grade classification performance across different image pre-processing strategies. † indicates the configuration used in the main manuscript.

| Method | Facial sign | | Pearson *r* | P-value | MAE | %MAE=0 | %MAE$\boldsymbol{\leq}$1 | %MAE$\boldsymbol{\leq}$2 |
| --- | --- | --- | --- | --- | --- | --- | --- | --- |
| Simply Resizing | | Forehead wrinkles | 0.80 | <0.0001 | 0.62 | 47.96 | 91.55 | 98.79 |
|  |  | Crow’s feet wrinkles | 0.83 | <0.0001 | 0.73 | 44.28 | 85.70 | 97.83 |
|  |  | Glabellar wrinkles | 0.82 | <0.0001 | 0.59 | 53.09 | 90.20 | 98.34 |
|  |  | Forehead pigmentation | 0.66 | <0.0001 | 0.50 | 56.21 | 94.39 | 99.70 |
|  |  | Cheek pigmentation | 0.74 | <0.0001 | 0.54 | 54.98 | 91.63 | 99.43 |
|  |  | Cheek pores | 0.53 | <0.0001 | 0.55 | 53.31 | 91.89 | **99.89** |
|  |  | Lip surface dryness | 0.16 | <0.0001 | 0.53 | 56.67 | 92.27 | 98.64 |
|  |  | Chin sagging | 0.72 | <0.0001 | 0.66 | 54.00 | 83.41 | 96.83 |
| Zero-Padding | | Forehead wrinkles | 0.77 | <0.0001 | 0.71 | 46.15 | 84.46 | 98.34 |
|  |  | Crow’s feet wrinkles | 0.81 | <0.0001 | 0.79 | 44.39 | 80.89 | 96.34 |
|  |  | Glabellar wrinkles | 0.78 | <0.0001 | 0.65 | 50.83 | 87.33 | 97.74 |
|  |  | Forehead pigmentation | 0.66 | <0.0001 | 0.51 | 54.70 | 94.70 | **100.00** |
|  |  | Cheek pigmentation | 0.76 | <0.0001 | 0.56 | 51.36 | 93.33 | **99.77** |
|  |  | Cheek pores | 0.48 | <0.0001 | **0.46** | **61.76** | 93.26 | 99.09 |
|  |  | Lip surface dryness | 0.34 | <0.0001 | 0.52 | 52.12 | **96.06** | **99.85** |
|  |  | Chin sagging | 0.75 | <0.0001 | 0.65 | 55.35 | 83.56 | 96.53 |
| BBox-based square crop^†^ | | Forehead wrinkles | **0.84** | <0.0001 | **0.52** | **55.81** | **92.91** | **99.10** |
|  |  | Crow’s feet wrinkles | **0.86** | <0.0001 | **0.62** | **52.63** | 87.76 | 98.05 |
|  |  | Glabellar wrinkles | **0.85** | <0.0001 | **0.55** | **54.60** | **92.01** | **99.10** |
|  |  | Forehead pigmentation | **0.79** | <0.0001 | **0.33** | **69.70** | **97.58** | 99.85 |
|  |  | Cheek pigmentation | **0.81** | <0.0001 | **0.45** | **60.41** | **94.68** | **99.66** |
|  |  | Cheek pores | **0.57** | <0.0001 | 0.48 | 57.99 | **94.63** | 99.77 |
|  |  | Lip surface dryness | **0.36** | <0.0001 | **0.45** | **60.61** | 94.39 | **99.85** |
|  |  | Chin sagging | **0.83** | <0.0001 | **0.53** | **60.33** | **90.05** | **97.29** |

**Table S6**. Comparison of facial-sign grade classification performance across different image input modalities. † indicates the configuration used in the main manuscript.

| Method | Facial sign | | Pearson r | P-value | MAE | %MAE=0 | %MAE$\boldsymbol{\leq}$1 | %MAE$\boldsymbol{\leq}$2 |
| --- | --- | --- | --- | --- | --- | --- | --- | --- |
| Gray-scale | | Forehead wrinkles | 0.80 | <0.0001 | 0.65 | 47.21 | 88.24 | **99.40** |
|  |  | Crow’s feet wrinkles | 0.84 | <0.0001 | 0.70 | 44.16 | **88.33** | **98.17** |
|  |  | Glabellar wrinkles | 0.80 | <0.0001 | 0.69 | 47.36 | 86.73 | 97.59 |
|  |  | Forehead pigmentation | 0.57 | <0.0001 | 0.56 | 56.21 | 89.09 | 98.33 |
|  |  | Cheek pigmentation | 0.71 | <0.0001 | 0.65 | 47.96 | 88.69 | 98.98 |
|  |  | Cheek pores | 0.49 | <0.0001 | 1.17 | 20.66 | 67.58 | 94.75 |
|  |  | Lip surface dryness | 0.14 | <0.0001 | 0.57 | 52.73 | 91.67 | 98.79 |
|  |  | Chin sagging | 0.72 | <0.0001 | 0.68 | 49.17 | 85.52 | 97.44 |
| Gray-scale + Histogram | | Forehead wrinkles | **0.84** | <0.0001 | 0.57 | 50.23 | **93.67** | **99.40** |
|  |  | Crow’s feet wrinkles | 0.84 | <0.0001 | 0.77 | 40.39 | 85.58 | 97.71 |
|  |  | Glabellar wrinkles | 0.84 | <0.0001 | 0.70 | 46.61 | 87.93 | 96.98 |
|  |  | Forehead pigmentation | 0.69 | <0.0001 | 0.47 | 59.39 | 94.70 | 99.09 |
|  |  | Cheek pigmentation | 0.73 | <0.0001 | 0.64 | 47.96 | 89.59 | 98.53 |
|  |  | Cheek pores | 0.48 | <0.0001 | **0.48** | **61.53** | 91.21 | 99.43 |
|  |  | Lip surface dryness | 0.17 | <0.0001 | 0.46 | 58.64 | **95.00** | **100.00** |
|  |  | Chin sagging | 0.74 | <0.0001 | 0.62 | 52.94 | 88.08 | **97.89** |
| RGB^†^ | | Forehead wrinkles | **0.84** | <0.0001 | **0.52** | **55.81** | 92.91 | 99.10 |
|  |  | Crow’s feet wrinkles | **0.86** | <0.0001 | **0.62** | **52.63** | 87.76 | 98.05 |
|  |  | Glabellar wrinkles | **0.85** | <0.0001 | **0.55** | **54.60** | **92.01** | **99.10** |
|  |  | Forehead pigmentation | **0.79** | <0.0001 | **0.33** | **69.70** | **97.58** | **99.85** |
|  |  | Cheek pigmentation | **0.81** | <0.0001 | **0.45** | **60.41** | **94.68** | **99.66** |
|  |  | Cheek pores | **0.57** | <0.0001 | **0.48** | 57.99 | **94.63** | **99.77** |
|  |  | Lip surface dryness | **0.36** | <0.0001 | **0.45** | **60.61** | 94.39 | 99.85 |
|  |  | Chin sagging | **0.83** | <0.0001 | **0.53** | **60.33** | **90.05** | 97.29 |

**Table S7**. Mean absolute error (MAE) across grade levels for the seven facial signs on the test dataset across four independent experiments (mean ± SD).

| Facial sign | Grade 0 | Grade 1 | | Grade 2 | Grade 3 | Grade 4 | Grade 5 | Grade 6 |
| --- | --- | --- | --- | --- | --- | --- | --- | --- |
| Forehead wrinkles | 0.50 ± 0.27 | 0.33 ± 0.21 | 0.22 ± 0.07 | | 0.25 ± 0.09 | 0.39 ± 0.18 | 0.44 ± 0.20 | 0.53 ± 0.31 |
| Crow’s feet wrinkles | 0.72 ± 0.15 | 0.09 ± 0.03 | 0.59 ± 0.16 | | 0.28 ± 0.05 | 0.67 ± 0.11 | 0.42 ± 0.20 | 0.32 ± 0.08 |
| Glabellar wrinkles | 0.27 ± 0.11 | 0.11 ± 0.04 | 0.50 ± 0.19 | | 0.51 ± 0.13 | 0.94 ± 0.07 | 0.42 ± 0.12 | 0.48 ± 0.26 |
| Forehead pigmentation | 0.15 ± 0.09 | 0.03 ± 0.03 | 0.27 ± 0.09 | | 0.39 ± 0.13 | 0.46 ± 0.18 | 0.17 ± 0.12 | - |
| Cheek pigmentation | 0.47 ± 0.15 | 0.07 ± 0.01 | 0.32 ± 0.14 | | 0.34 ± 0.04 | 0.72 ± 0.20 | 0.58 ± 0.39 | - |
| Cheek pores | 0.03 ± 0.05 | 0.39 ± 0.13 | 0.17 ± 0.03 | | 0.87 ± 0.14 | 0.64 ± 0.40 | 0.00 ± 0.00 | - |
| Lip surface dryness | 1.61 ± 0.12 | 0.91 ± 0.09 | 0.14 ± 0.06 | | 0.77 ± 0.11 | 1.50 ± 0.23 | - | - |
| Chin sagging | 0.09 ± 0.06 | 0.62 ± 0.37 | 0.48 ± 0.32 | | 0.58 ± 0.12 | 0.23 ± 0.10 | 0.07 ± 0.12 | - |

**Table S8**. TRIPOD-AI Reporting Checklist for the Present Study.

| **Section/Topic** | **Item** | **Checklist item** | **Reported on page** |
| --- | --- | --- | --- |
| **TITLE** | | | |
| Title | 1 | Identify the study as developing or evaluating the performance of a multivariable prediction model, the target population, and the outcome to be predicted | p. 1-2 |
| **ABSTRACT** | | | |
| Abstract | 2 | See TRIPOD+AI for Abstracts checklist | p. 2 |
| **INTRODUCTION** | | | |
| Background | 3a | Explain the healthcare context (including whether diagnostic or prognostic) and rationale for developing or evaluating the prediction model, including references to existing models | p. 3 |
|  | 3b | Describe the target population and the intended purpose of the prediction model in the context of the care pathway, including its intended users (e.g., healthcare professionals, patients, public) | p. 2-3, 10-11 |
|  | 3c | Describe any known health inequalities between sociodemographic groups | N/A |
| Objectives | 4 | Specify the study objectives, including whether the study describes the development or validation of a prediction model (or both) | p. 2-3 |
| **METHODS** | | | |
| Data | 5a | Describe the sources of data separately for the development and evaluation datasets (e.g., randomised trial, cohort, routine care or registry data), the rationale for using these data, and representativeness of the data | p. 4-7 |
|  | 5b | Specify the dates of the collected participant data, including start and end of participant accrual; and, if applicable, end of follow-up | p. 7 |
| Participants | 6a | Specify key elements of the study setting (e.g., primary care, secondary care, general population) including the number and location of centres | p. 3-4 |
|  | 6b | Describe the eligibility criteria for study participants | p. 3-4 |
|  | 6c | Give details of any treatments received, and how they were handled during model development or evaluation, if relevant | N/A |
| Data preparation | 7 | Describe any data pre-processing and quality checking, including whether this was similar across relevant sociodemographic groups | p. 4 |
| Outcome | 8a | Clearly define the outcome that is being predicted and the time horizon, including how and when assessed, the rationale for choosing this outcome, and whether the method of outcome assessment is consistent across sociodemographic groups | p. 4-5; Table 2 |
|  | 8b | If outcome assessment requires subjective interpretation, describe the qualifications and demographic characteristics of the outcome assessors | p. 4-5 |
|  | 8c | Report any actions to blind assessment of the outcome to be predicted | p. 5 |
| Predictors | 9a | Describe the choice of initial predictors (e.g., literature, previous models, all available predictors) and any pre-selection of predictors before model building | p. 4-5 |
|  | 9b | Clearly define all predictors, including how and when they were measured (and any actions to blind assessment of predictors for the outcome and other predictors) | p. 4-5 |
|  | 9c | If predictor measurement requires subjective interpretation, describe the qualifications and demographic characteristics of the predictor assessors | N/A |
| Sample size | 10 | Explain how the study size was arrived at (separately for development and evaluation), and justify that the study size was sufficient to answer the research question. Include details of any sample size calculation | p.3-4; Table 1 |
| Missing data | 11 | Describe how missing data were handled. Provide reasons for omitting any data | N/A |
| Analytical methods | 12a | Describe how the data were used (e.g., for development and evaluation of model performance) in the analysis, including whether the data were partitioned, considering any sample size requirements | p. 6-7; Table 7 |
|  | 12b | Depending on the type of model, describe how predictors were handled in the analyses (functional form, rescaling, transformation, or any standardisation). | p. 4; Figure S1 |
|  | 12c | Specify the type of model, rationale, all model-building steps, including any hyperparameter tuning, and method for internal validation | p. 5-7 |
|  | 12d | Describe if and how any heterogeneity in estimates of model parameter values and model performance was handled and quantified across clusters (e.g., hospitals, countries). | N/A |
|  | 12e | Specify all measures and plots used (and their rationale) to evaluate model performance (e.g., discrimination, calibration, clinical utility) and, if relevant, to compare multiple models | p. 7-9; Tables 5-10; Figure 4 |
|  | 12f | Describe any model updating (e.g., recalibration) arising from the model evaluation, either overall or for particular sociodemographic groups or settings | N/A |
|  | 12g | For model evaluation, describe how the model predictions were calculated (e.g., formula, code, object, application programming interface) | N/A |
| Class imbalance | 13 | If class imbalance methods were used, state why and how this was done, and any subsequent methods to recalibrate the model or the model predictions | p. 5-6 |
| Fairness | 14 | Describe any approaches that were used to address model fairness and their rationale | N/A |
| Model output | 15 | Specify the output of the prediction model (e.g., probabilities, classification). Provide details and rationale for any classification and how the thresholds were identified | p. 5-6 |
| Training versus evaluation | 16 | Identify any differences between the development and evaluation data in healthcare setting, eligibility criteria, outcome, and predictors | p. 6, 8-9 |
| Ethical approval | 17 | Name the institutional research board or ethics committee that approved the study and describe the participant-informed consent or the ethics committee waiver of informed consent | p. 2-3 |
| **OPEN SCIENCE** | | | |
| Funding | 18a | Give the source of funding and the role of the funders for the present study | p. 1 |
| Conflicts of interest | 18b | Declare any conflicts of interest and financial disclosures for all authors | p. 1 |
| Protocol | 18c | Indicate where the study protocol can be accessed or state that a protocol was not prepared | N/A |
| Registration | 18d | Provide registration information for the study, including register name and registration number, or state that the study was not registered | N/A |
| Data sharing | 18e | Provide details of the availability of the study data | N/A |
| Code sharing | 18f | Provide details of the availability of the analytical code | N/A |
| **PATIENT & PUBLIC INVOLVEMENT** | | | |
| Patient & Public Involvement | 19 | Provide details of any patient and public involvement during the design, conduct, reporting, interpretation, or dissemination of the study or state no involvement. | N/A |
| **RESULTS** | | | |
| Participants | 20a | Describe the flow of participants through the study, including the number of participants with and without the outcome and, if applicable, a summary of the follow-up time. A diagram may be helpful. | P.6-7; Table 7 |
|  | 20b | Report the characteristics overall and, where applicable, for each data source or setting, including the key dates, key predictors (including demographics), treatments received, sample size, number of outcome events, follow-up time, and amount of missing data. A table may be helpful. Report any differences across key demographic groups. | p. 7; Tables 1, 3, 7, 8 |
|  | 20c | For model evaluation, show a comparison with the development data of the distribution of important predictors (demographics, predictors, and outcome). | Tables 5, 8, 9 |
| Model development | 21 | Specify the number of participants and outcome events in each analysis (e.g., for model development, hyperparameter tuning, model evaluation) | p. 6-7; Table 7 |
| Model specification | 22 | Provide details of the full prediction model (e.g., formula, code, object, application programming interface) to allow predictions in new individuals and to enable third-party evaluation and implementation, including any restrictions to access or re-use | N/A |
| Model performance | 23a | Report model performance estimates with confidence intervals, including for any key subgroups (e.g., sociodemographic). Consider plots to aid presentation. | Tables 5, 6, 9 |
|  | 23b | If examined, report results of any heterogeneity in model performance across clusters. | N/A |
| Model updating | 24 | Report the results from any model updating, including the updated model and subsequent performance | N/A |
| **DISCUSSION** | | | |
| Interpretation | 25 | Give an overall interpretation of the main results, including issues of fairness in the context of the objectives and previous studies | p. 9-10 |
| Limitations | 26 | Discuss any limitations of the study (such as a non-representative sample, sample size, overfitting, missing data) and their effects on any biases, statistical uncertainty, and generalizability | p. 10-11 |
| Usability of the model in the context of current care | 27a | Describe how poor quality or unavailable input data (e.g., predictor values) should be assessed and handled when implementing the prediction model | p. 10 |
|  | 27b | Specify whether users will be required to interact in the handling of the input data or use of the model, and what level of expertise is required of users | N/A |
|  | 27c | Discuss any next steps for future research, with a specific view to applicability and generalizability of the model | p. 10-11 |

# Figures

|  |  |
| --- | --- |
| (a) Original rectangular regional image | (b) Resulting square image after bounding box–based cropping |

**Figure S1**. Representative example of the bounding box–based square cropping procedure used to convert elongated facial-region patches into standardized square inputs for AI analysis.

**Figure S2**. Overall architecture of the CoAtNet-4 model used as the backbone for facial skin analysis.
